# Intrahaplotypic Variants Differentiate Complex Linkage Disequilibrium within Human MHC Haplotypes

**DOI:** 10.1038/srep16972

**Published:** 2015-11-23

**Authors:** Tze Hau Lam, Matthew Zirui Tay, Bei Wang, Ziwei Xiao, Ee Chee Ren

**Affiliations:** 1 Singapore Immunology Network, A*STAR, 8A Biomedical Grove, #03-06 Immunos, Singapore 138648, Singapore; 2 Department of Microbiology, Yong Loo Lin School of Medicine, National University of Singapore, 5 Science Drive 2, MD4 Level 5, Singapore 117597, Singapore; 3 Department of Molecular Genetics and Microbiology, Duke University, Durham, NC 27710, USA

## Abstract

Distinct regions of long-range genetic fixation in the human MHC region, known as conserved extended haplotypes (CEHs), possess unique genomic characteristics and are strongly associated with numerous diseases. While CEHs appear to be homogeneous by SNP analysis, the nature of fine variations within their genomic structure is unknown. Using multiple, MHC-homozygous cell lines, we demonstrate extensive sequence conservation in two common Asian MHC haplotypes: A33-B58-DR3 and A2-B46-DR9. However, characterization of phase-resolved MHC haplotypes revealed unique intra-CEH patterns of variation and uncovered 127 single nucleotide variants (SNVs) which are missing from public databases. We further show that the strong linkage disequilibrium structure within the human MHC that typically confounds precise identification of genetic features can be resolved using intra-CEH variants, as evidenced by rs3129063 and rs448489, which affect expression of *ZFP57*, a gene important in methylation and epigenetic regulation. This study demonstrates an improved strategy that can be used towards genetic dissection of diseases.

The human Major Histocompatibility Complex (MHC) region on chromosome 6p21.3 is the most gene-dense region in the human genome^1^,^2^, containing gene families essential to the immune system, and is important for the study of population history and demographic events^3^,^4^. In addition to its high gene density, linkage disequilibrium (LD) is one of the distinctive genetic features of the MHC region. The distribution of LD is not homogenous across the MHC region, but is consistently maintained in the regions between HLA-C and HLA-B, as well as HLA-DRB1 and HLA-DQA1, with relatively low LD found in the intervening regions. This results in the delineation of the MHC region into blocks of LD haplotypes according to the underlying MHC allelic haplotype background^5^,^6^. Each block, defined by its linked MHC alleles, is known as the MHC haplotype. Within the human population, there are a number of MHC haplotypes called conserved extended haplotypes (CEHs), which exhibit distinctively long stretches of nearly identical sequences where recombination events appear to be unusually low or absent over megabases of DNA^7^,^8^. It has been estimated that approximately 80% of haplotypes achieving >0.5% frequency in a population may be CEHs^7^. One of the most well-studied conserved extended haplotypes is the HLA A1-B8-DR3-DQ2 haplotype, which extends 4.7 Mb from the *TRIM27* locus to the *SYNGAP1* locus^9^, and is found at relatively high frequencies (9%) in the northern European population^10^. The presence of CEHs has also been reported in other populations, including the A24-C12-B52-DR15 and A33-C14-B44-DR13 CEHs, which are commonly found in the Japanese population^11^. In addition to their unique genomic traits, CEHs are of particular interest as a result of the known associations of common CEHs with numerous diseases^12^,^13^. For instance, the A1-B8-DR3-DQ2 haplotype is a risk factor for type 1 diabetes, systemic lupus erythematosus, rheumatoid arthritis, and IgA deficiency, amongst others^14^,^15^,^16^. Despite the strength of these risk associations, genetic dissections of the causal gene or loci in CEHs remain incomplete because of the extensive sequence similarity in CEHs, and, more importantly, because the extent of intra-haplotypic variation within the conserved region is not well understood.

Attempts to quantify the level of polymorphism within MHC haplotypes have been carried out on eight European MHC haplotypes using bacterial artificial chromosome cloning (BACs) and shotgun sequencing^17^,^18^. Among these, only the MHC haplotypes of PGF (A3-B7-DR15-DQ6) and COX (A1-B8-DR3-DQ2) were sequenced completely and the MHC reference sequence of PGF was incorporated into the mosaic NCBI Build 37.2 reference sequence^9^. Each of these haplotypes was assembled into a haploid sequence from a single consanguineous cell line using BAC-derived sequences; however, there is no information on intra-haplotypic variation for any one particular MHC haplotype. Hence, the characteristic features of these haplotypes could not be determined. Subsequent studies employed target region and next generation re-sequencing approaches to interrogate variations residing within the MHC region^19^,^20^, but the cell lines investigated were either HLA heterozygous or did not exhibit CEH characteristics in the MHC genomic region. Partial re-sequencing studies were also performed to examine the sequence conservation in the CEHs^21^,^22^. Unfortunately, less than 20% of the conserved region was sequenced in these studies, and the authors were therefore unable to explore the scope of variation in CEHs definitively.

Here, we report the characterization of two Asian conserved extended haplotypes, A*33:03-C*03:02-B*58:01-DRB1*03:01 (A33-B58-DR3) and A*02:07-C*01:02-B*46:01-DRB1*09:01 (A2-B46-DR9), which are present in the Singapore Chinese population at relatively high frequencies (approximately 7% and 6%, respectively)^23^,^24^. In contrast to earlier studies, which only examined one representative of a particular MHC haplotype, we compared three unrelated individuals homozygous for each CEH using whole genome sequencing data. The data, which provides a nucleotide-resolution view of these prominent Asian CEHs, allows the assessment of intra-CEH conservation and variation in the extended MHC region. We also provide evidence of the impact of intra-CEH variants, identified within the conserved region of the CEH, at the level of gene expression. Finally, through inter-haplotypic sequence comparison, we reveal an evolutionary conservation of the HLA-DR3 haplotype between two common CEHs from different ethnic backgrounds, indicative of a common shared ancestor.

## Results

### Identification of novel Asian Conserved Extended Haplotypes (CEHs)

**Figure 1:**
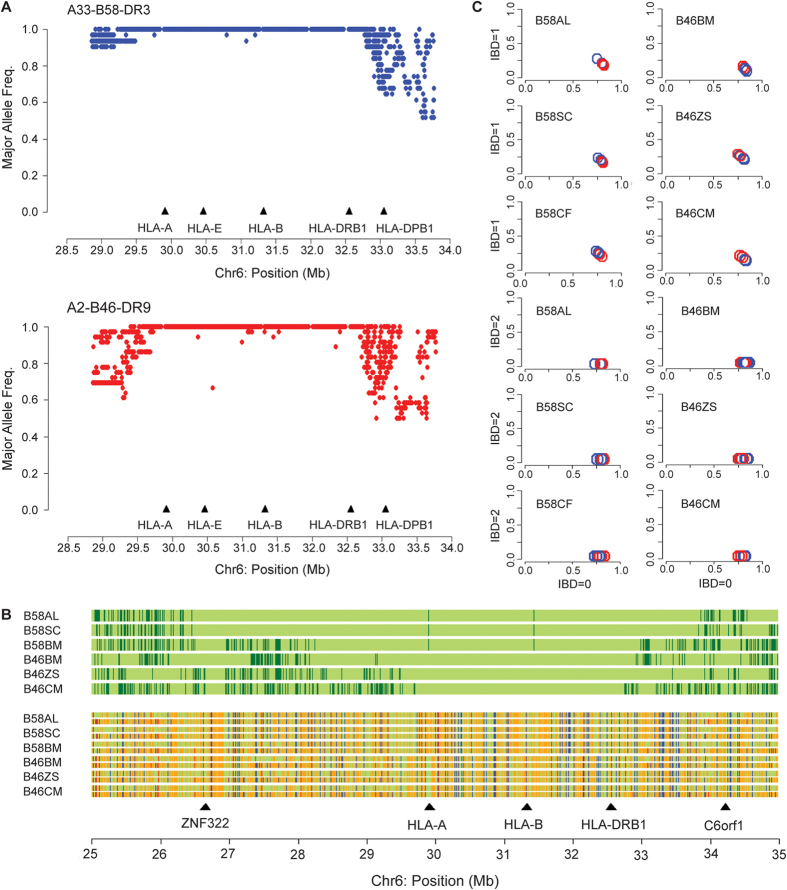
Conserved extended haplotype in A33-B58-DR3 and A2-B46-DR9 across the MHC region. **(A)** Major allele frequency plots for 1877 SNPs derived from independent A33-B58-DR3 (n = 31) and A2-B46-DR9 (n = 36) chromosomes. **(B)** SNP alignment of six HLA homozygous individuals carrying a specific haplotype. The SNP alignment includes the genotype status of each SNP (green vertical bars indicate a homozygous SNP call and dark green vertical bars indicate a heterozygous SNP call) and the SNP allelic call with reference to its position (green = adenine, red = cytosine, orange = guanine, and blue = thymine). 10125 SNP markers are involved in the alignment. **(C)** Identity-by-descent (IBD) analysis. Pairwise IBD plots (IBD = 1 vs IBD = 0 and IBD = 2 vs IBD = 0) of a reference individual with the other respective individuals. The blue circle indicates pairwise analysis of individuals carrying A33-B58-DR3 while the red circle indicates pairwise analysis of individuals carrying A2-B46-DR9.

Using an MHC-specific screening panel of 1877 single-nucleotide polymorphisms (SNPs) in combination with HLA typing data of 247 unrelated Chinese individuals, two distinct patterns of the extended MHC region HLA-A33-B58-DR3 (n = 31) and HLA-A2-B46-DR9 (n = 36) were clearly identified (Fig. 1A; Supplementary Fig. S1). Three samples of each haplotype with homozygous HLA types were identified,A33-B58-DR3 (B58AL, B58SC, B58CF) and A2-B46-DR9 (B46BM, B46ZS, B46CM), from which genomic DNA was purified and analysed using the Illumina Human 1 M-Duo BeadChip SNP array. After quality filtering, 7509 SNPs were found to be informative within the extended MHC region (28.5 Mb to 33.5 Mb). These SNP markers were then subjected to Runs of Homozygosity (ROH) analysis to assess the SNP homozygosity profile in each cell line. Regions of conservation were identified for each sample based on levels of homozygosity within the extended MHC region (Supplementary Table S1). Homozygosity and intra-CEH conservation were found not only at the HLA loci, but also across the extended MHC region (Fig. 1B). For A33-B58-DR3, at least 99.5% of SNPs were found to be homozygous across a 4.6-Mb region, and the genotype calls for these homozygous SNPs were consistent in all three A33-B58-DR3 cell lines. Likewise, for A2-B46-DR9, 99.7% of the encompassing SNPs genotyped as homozygous across all three A2-B46-DR9 cell lines were conserved across a 3.1-Mb region. To verify that the large segments of conservation observed within the MHC region were not a result of undetected familial relatedness among these individuals, an identity-by-descent (IBD) analysis was performed using the whole-genome SNP information of all six individuals. No evidence of relatedness was found (Fig. 1C), confirming that the high level of conservation existing between these individuals cannot be attributed to familial relationships. Taken together, these analyses reveal that, for both the A33-B58-DR3 and A2-B46-DR9 MHC haplotypes, linkage of the HLA alleles is not restricted to the HLA loci, and that mega-bases of flanking genomic segments are inherited together in strong linkage disequilibrium.

### Fine-scale mapping of A33-B58-DR3 and A2-B46-DR9 CEHs using deep-sequencing

The use of high-density SNP typing (average 1/665 bp) showed that CEHs of HLA-identical, unrelated individuals appeared to be indistinguishable. However, it is not yet known if this conservation is maintained at the nucleotide level. To address this question, the HLA homozygous cell lines, B58AL, B58SC, B58CF, B46BM, B46ZS, and B46CM, were subjected to whole-genome sequencing (WGS) using the Complete Genomics (CG) platform. Raw reads were processed by the Complete Genomics Standard Sequencing Pipeline 2.0, and assembled according to the Genome Reference Consortium Human genome build 37 (GRCh37)^25^. For each sequenced genome, the mean coverage per bp was at least 37.13 times, covering no less than 94.18% of the extended MHC region (28.5 Mb to 33.5 Mb) (Supplementary Fig. S2 and Supplementary Table S2).

To assess the quality of data generated from the CG platform, genotype calls between 25 and 35 Mb from the Illumina Human 1 M-Duo BeadChip SNP array were compared with the CG sequencing data. The results showed a high concordance rate (99.5% for A33-B58-DR3 and 99.6% for A2-B46-DR9) between SNP genotyping and the CG data (Supplementary Table S3). A random sample of 48 call points from among the discordant data was selected for validation by PCR re-sequencing. Of these, 45/48 were found to be consistent with the CG data, while 3/48 agreed with the SNP genotyping array data (Supplementary Table S4 - S5), indicating that, in general, the CG sequencing platform delivers higher call accuracy than the SNP genotyping platform.

Next, the range of genomic conservation in each sample was assessed with nucleotides from the 25–35 Mb region of chromosome 6 binned into windows of 5 kb, and the number of homozygous or heterozygous RefSeq single nucleotide variation (SNV) calls within each bin were examined. Stretches of homozygosity were defined to be regions with no more than four consecutive windows with a zygosity SNV ratio (number of homozygous SNVs against total number of SNVs in a given window) of less than 0.95 (Supplementary Fig. S3). The resulting conserved region in each genome coincided with the region determined using the SNPs genotyping platform (Supplementary Table S1). Within the conserved segment boundaries, CG data showed at least 99.99% homozygosity in all samples, with, on average, 8.33 heterozygous calls per 100 kb in the A33-B58-DR9 haplotype samples and 4.89 heterozygous calls per 100 kb in the A2-B46-DR3 haplotype samples. Notably, occurrences of heterozygous variants were randomly spread across the conserved MHC region even though high density SNP profiling had indicated previously that this genomic region was homozygous. This important feature of fine single nucleotide variants has not been observed in previous studies of CEHs, including high-density SNP analyses, which lacked the necessary resolution.

### Intra-CEH conservation and variation

**Figure 2:**
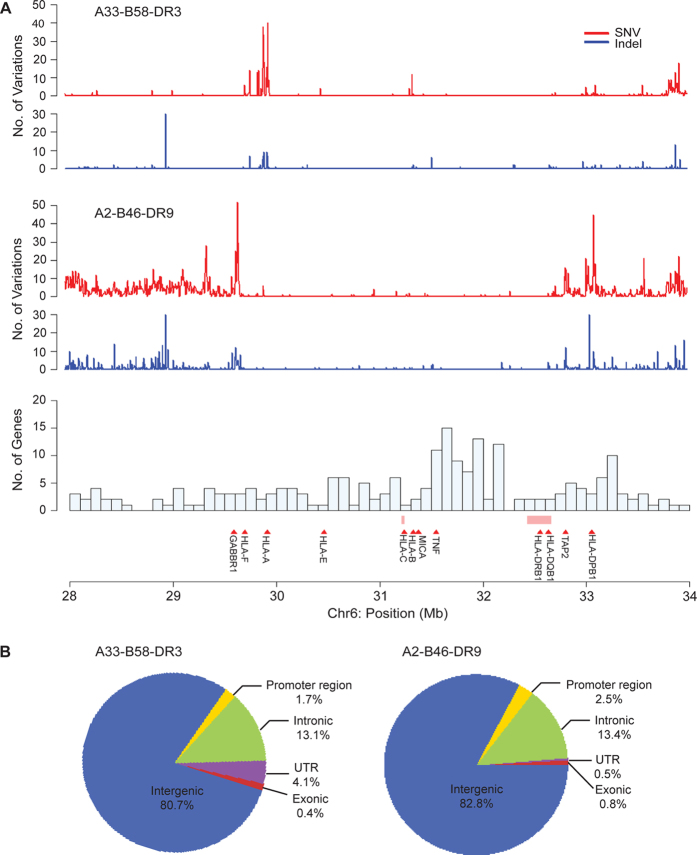
Distribution of intra-CEH variations across the MHC region. **(A)** Each data point on the plot represents the number of SNV counts (red) and the number of indel counts (blue) in a non-overlapping 5-kb window. The number of variations for each haplotype was derived from the comparison of six haploid chromosomes at every possible nucleotide position across the MHC region. The pink bars indicate regions where the sequences are ambiguous and nucleotide positions within these regions are not compared. **(B)** Annotation of intra-CEH variants based on genomic features found in the NCBI RefSeq Build 37.2 gene annotation.

The availability of homozygous MHC CEHs offered the opportunity to characterize the extent of intra-CEH variation and conservation. The CG platform generated unambiguous sequences of 4,135,945 bp, representative of the A33-B58-DR3 haplotype, and 2,720,646 bp, representative of the A2-B46-DR9 haplotype. The degree of intra-CEH variation was found to be exceptionally low (Fig. 2A); 290 SNVs and 52 indels (nucleotide insertions or deletions) were identified in the A33-B58-DR3 haplotype, while 238 SNVs and 51 indels were observed in the A2-B46-DR9 haplotype (Table 1). The estimated nucleotide diversity value (π) between the A33-B58-DR3 haploid sequences and A2-B46-DR9 haploid sequences was 7.08 × 10^−5^ and 8.75 × 10^−5^, respectively. These values are at least 38-fold lower than that found between the two common European MHC haplotypes, PGF and COX (3.4 × 10^–3^)^17^, and at least 5-fold lower than the nucleotide diversity between any two haplotypes across the human genome^26^,^27^, indicative of extremely low nucleotide diversity in the A33-B58-DR3 and A2-B46-DR9 MHC haplotypes. Closer inspection revealed that the majority of intra-CEH variations in each MHC haplotype were localized to a single region. For example, spikes of variation localized to a 120-kb region, covering the *ZFP57* and *HLA-F* genes (chr6:29,600,360–29,721,396), were found in the A2-B46-DR9 haplotype but not in the A33-B58-DR3 haplotype (Supplementary Fig. S4A). These variations comprised >70% (171/238) of the total A2-B46-DR9 intra-CEH SNVs. Similarly, elevated numbers of A33-B58-DR3 intra-CEH variations accounting for 90% (262/293) of SNVs were observed over a 240-kb region covering the *HLA-A* gene (chr6: 29,733,502–29,971,973), while the number of intra-CEH variations in the A2-B46-BR9 over this region was distinctly lower (Supplementary Fig. S4B).

Using the NCBI RefSeq Build 37.2 gene annotation, intra-CEH variations were grouped according to specific genomic features (Table 1). The majority of the variants (~80%) resided in the intergenic region, while less than 0.8% of all variants were located in the exonic region (Fig. 2B), indicating a high degree of coding sequence (CDS) conservation. CDS variants were only found in two genes (MOG, NOTCH4) in the A2-B46-DR9 haplotype and one gene (HLA-A) in the A33-B58-DR9 haplotype (Supplementary Table S6). Surprisingly, the single CDS variant in the A33-B58-DR3 haplotype was a missense mutation in exon 7 of HLA-A. To validate this, the HLA-A exon 7 (position 29,913,037) missense variant was cloned and re-sequenced, and its presence was confirmed in all three A33-B58-DR3 samples (Supplementary Fig. S5–S7). Within this 479-bp cloned fragment, 13 heterozygous and 15 homozygous RefSeq variants were found to be in agreement with the CG data (Supplementary Table S7). The two CDS variants in A2-B46-DR9 were verified by PCR resequencing (Supplementary Table S8). In total, 77 novel SNV variants within the A33-B58-DR3 samples and 50 novel SNV variants within the A2-B46-DR9 samples were identified that were not annotated in dbSNP build 132, the 1000 Genome Project, or the International HapMap Project (Supplementary Table S9-S10). These novel SNPs may provide valuable additional markers that can be utilised in disease-association studies.

### Effect of intra-CEH variation on regulation of ZFP57 gene expression

**Figure 3:**
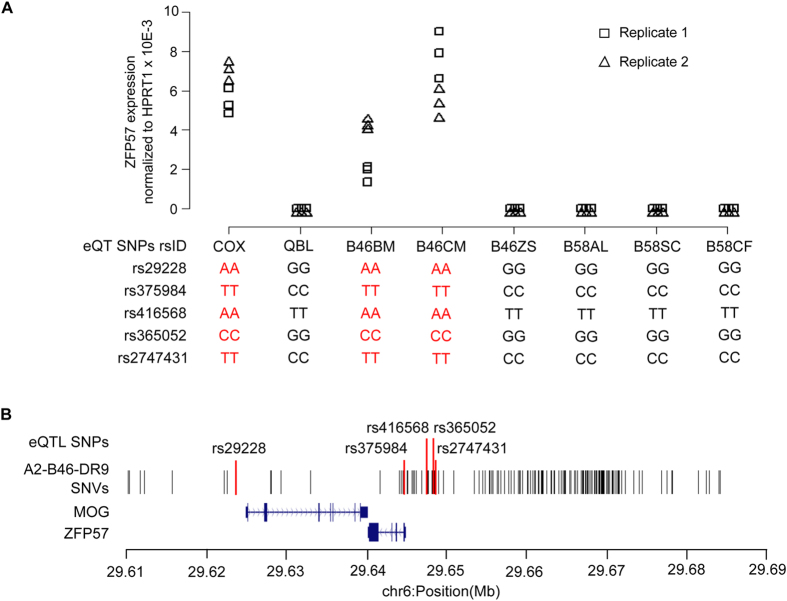
Association of A2-B46-DR9 intra-CEH variants with *ZFP57* expression. **(A)** qRT-PCR was performed to determine the mRNA level of *ZFP57* in COX, QBL, B46BM, B46ZS, B46CM, B58AL, B58SC, and B58CF cell lines. Experiments were carried out in triplicate for each biological replicate. Triangles indicate quantitative expression derived from biological replicate 1, while squares indicate quantitative expression derived from biological replicate 2. The genotype of the eQTL SNPs in each cell line are shown below the plot. **(B)** Distribution of the 202 A2-B46-DR9 intra-CEH variants across chr6:29.60–29.69(Mb).

To determine whether fine nucleotide variations within MHC CEHs have functional effects on gene expression, we analysed a known rs29228 SNP (chr6: 29,623,739) that was reported to exert a cis-acting effect on the expression level of the Zinc Finger Protein 57 homolog (*ZFP57*) located 16.43 kb centromeric to rs29228^28^. It was noted that carriers of the “AA” but not the “GG” genotype of rs29228 would support expression of *ZFP57*. A more recent study revealed four additional eQTL SNPs (chr6:29,644,502 - rs375984, chr6:29,647,628 - rs416568, chr6:29,648,398 - rs365052, and chr6:29,648,564 - rs2747431), located in the *ZFP57* introns and promoter region, that were associated with the expression of *ZFP57*^29^. Interestingly, intra-CEH variants were present at these SNP positions in the A2-B46-DR9 haplotype but not the A33-B58-DR3 haplotype. Reverse transcription quantitative PCR was performed to evaluate the *ZFP57* mRNA levels in B58AL, B58SC, B58CF, B46BM, B46ZS, B46CM, as well as COX and QBL. Noticeably, the COX, B46BM, and B46CM (both A2-B46-DR9) cell lines which possess the “A” allele of rs29228, “T” allele of rs375984, “A” allele of rs416568, “C” allele of rs365052, and “T” allele of rs2747431 expressed *ZFP57* while B46ZS (A2-B46-DR9), B58AL, B58SC, B58CF (all A33-B58-DR3), and QBL which possess the alternate allele at these positions have no *ZFP57* expression (Fig. 3A).

**Figure 4:**
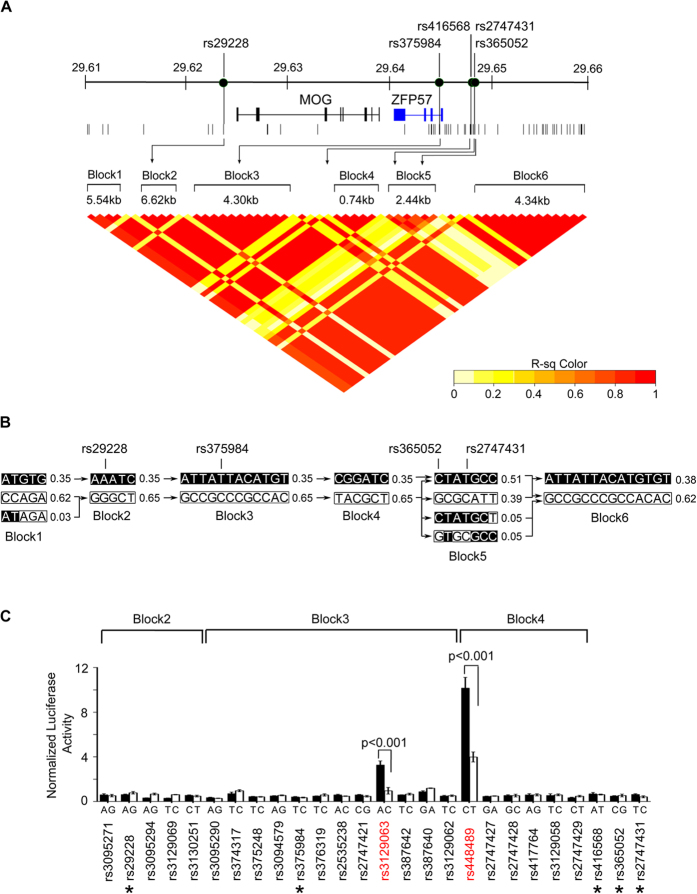
Mapping of regulatory elements to ZFP57 expression. (**A**) Linkage disequilibrium (LD) plot for region chr6:29.61 – 26.66(Mb) based on the r^2^ calculation of 163 A2-B46-DR9 intra-CEH variants/SNPs in 105 unrelated Southern Han Chinese individuals (1000 Genome Project). LD block is defined as a block of SNPs with all pairwise r^2^ value >0.8. **(B)** Haplotypes found in each LD block. Population haplotype frequency is shown at the side of each haplotype. Connecting lines between blocks indicate crossover to the next block. Block in black indicates allele of variant associated with ZFP57 expression while white indicates allele associated with non ZFP57 expression. **(C)** Luciferase activity for alleles of each intra-CEH variants in LD blocks 2 to 4. Reporter plasmids carrying the fragment sequence of each unique allele of a certain variant were co-transfected with Renilla control vector into HT1080 cells. The luciferase activity readout was normalized to Renilla luciferase activity and the representative results from three independent experiments are shown. Variants with significant differential allelic luciferase activity (P < 0.01, t-test) are highlighted in red. Reported eQTL SNPs are marked with *.

The presence of eQTL SNPs associated with *ZFP57* indicates a possible regulatory role of polymorphic sites in the genomic region. To map putative regulatory variants to *ZFP57*, we examined the intra-CEH sequence variation in the A2-B46-DR9 cell lines over the 90-kb genomic region encompassing the *ZFP57* gene (chr6:29,600,000–29,690,000). A total of 202 A2-B46-DR9 intra-haplotypic SNVs were found within this genomic segment, the majority of which were localised to the 5′ end of the *ZFP57* gene (Fig. 3B). Of these, 170 variants from the B46ZS cell line matched with all three A33-B58-DR3 cell lines, identifying these sites as potential regulatory variant candidates, although some variation was noted across this 90-kb region between the sequences of B46ZS and the A33-B58-DR3 haplotype (Supplementary Fig. S8). Next, we proceeded to establish the LD structure of this 90-kb region using the genetic information of 163/170 intra-CEH variants from a Southern Han Chinese population of 105 unrelated individuals (1000 Genomes Project)^30^,^31^. From the LD map, we identified a tight LD region, consisting of a cluster of six LD blocks between chr6:29.61-29.66(Mb) encompassing the eQTL SNPs as well as the *MOG* and *ZFP57* genes (Fig. 4A). Detailed analysis of these LD blocks revealed two distinct groups of haplotypes in fixed frequency that are in linkage with rs29928 and rs375984 in LD blocks 2 to 4; where one group contained alleles of eQTL SNPs associated with ZFP57 expression, while the other group contained the alternate alleles associated with a lack of ZFP57 expression (Fig. 4B). The rs416568 SNP was found to be in a region of low LD between LD blocks 3 and 4, and both rs365052 and rs2747431 were found in the heterogeneous group of haplotypes in LD block 5, where there is no clear allelic association of the eQTLs SNPs with *ZFP57* expression. These results suggest that the regulatory variants of *ZFP57* were likely to be within LD blocks 2 to 4.

To test the regulatory effects of variants, 26 promoter-luciferase reporter constructs representing individual intra-CEH variants of A2-B46-DR9 were generated. These included nine variants (Supplementary Fig. S9A) outside of the three LD blocks, but within chr6:29.61-29.69(Mb), that mapped to regions with elevated levels of H3K4Me1 activity based on the histone modification data of B-LCL GM12878^32^. Of the 26 sites tested, rs3129063 (allele “A”) and rs448489 (allele “C”) exhibited significant increases in luciferase activity compared with their alternate alleles (Fig. 4C and Supplementary Fig. S9B). Both variants, ~2 kb apart, fall within the promoter region of *ZFP57,* denoting a cis control feature in the expression of *ZFP57*. These results provide evidence that minor nucleotide differences within a specific CEH can regulate gene expression differentially.

### Inter-haplotype evaluation reveals non-random genetic variation across the MHC region

**Figure 5:**
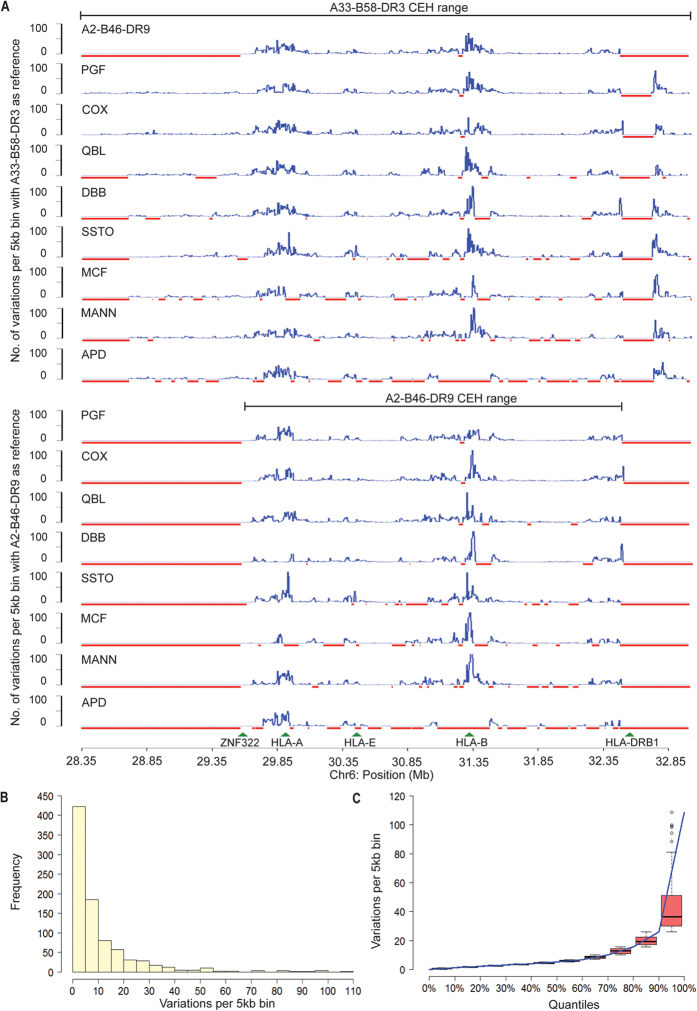
Pairwise inter-haplotypic variation across the MHC region. (**A**) A33-B58-DR3 and A2-B46-DR9 haplotype sequences were compared with each of the eight common European-descent MHC haplotypes (PGF, COX, QBL, DBB, SSTO, MCF, MANN, APD). The variation counts were binned into non-overlapping 5-kb windows. The red bars indicate gaps in the sequence of the haplotypes. (**B**) Frequency histogram of the number of variations for each 5-kb window. **(C)** Cumulative distribution for the number of variations per 5-kb bin across each 10% quantile interval.

Next, the A33-B58-DR3 and A2-B46-DR9 CEHs were compared against eight haplotypes of European origin: PGF, COX, QBL, APD, DBB, MANN, MCF, and SSTO^18^. The derived consensus haploid sequences for the A33-B58-DR3 and A2-B46-DR9 haplotypes were aligned pairwise with each of the eight European MHC haploid data. We observed that the chromosomal regions around HLA-A (29.6–30.0 Mb) and HLA-C – HLA-B (31.25–31.5 Mb) formed distinct regions of increased variation (Fig. 5A). Because the various haplotypes compared have different HLA-A, -B and -C alleles, divergence at these loci is expected. However, although the HLA loci alone are less than 12 kb in length, the peaks of variation are more than 200 kb in length. It has been reported that flanking regions of the HLA loci, extending up to 200 kb, can be strongly linked to the associated HLA alleles^6^,^24^. A comparison of the genomic features of the variants of the two Asian CEH haplotypes with PGF and COX, for which complete MHC genomic information is available, was performed. Unsurprisingly, the level of inter-haplotype variation was significantly higher than the level of intra-CEH variation (Table 1). Likewise, an elevated percentage (~2.2%) of inter-haplotype variation occurred in the gene-coding region. The estimated π between the Asian haplotypes was 2.70 × 10^−3^, whereas the π values between the Asian and the European haplotypes ranged from 2.11 × 10^−3^ to 2.55 × 10^−3^, indicating similar levels of variation in the MHC region between the Asian haplotypes and the Asian and European haplotypes.

To examine the patterns of inter-haplotype variation, the 28.35–32.95-Mb region was binned into windows of 5 kb in length, and a frequency histogram of the number of variations for each window was plotted (Fig. 5B). While most windows showed relatively low levels of variation, a few windows displayed extremely high levels (Fig. 5C). The top 10% of windows with the most variants contained 28–109 variants each, several-fold greater than the MHC region-wide average of 5.39, demonstrating that variation across the MHC region among the different haplotypes is generally low except in regions surrounding the HLA genes. Next, we identified 30-kb windows containing <3 variants or >15 variants to mark out regions of low and high variation, respectively (Supplementary Table 11). Surprisingly, a cluster of low-variation regions was observed surrounding the RCCX region, which included the C2 and RAGE loci. This may indicate conservation of these essential components of the innate immune system. The regions containing the highest amounts of variation were seen in the class I and class II loci and their neighbouring regions. The level of variation in these regions was remarkable (on average 8-fold that of MHC region-wide variation).

The RCCX region within chr6:31,939,646–32,077,151 is a common, multi-allelic copy number variation locus. The number of modules and type of C4 complement genes within the RCCX regions vary between individuals, and gene dosage of C4A and C4B has been associated with various disorders. For instance, lower levels of C4A have been associated with susceptibility to systemic lupus erythematosis^33^, while lower levels of C4B have been associated with increased rates of acute myocardial infection and stroke^34^. To identify the number and type of RCCX modules associated with each of the Asian haplotypes, we interrogated the RCCX region of each sample using a SYBR Green real-time PCR assay with primers specific for C4A, C4B, C4L, C4S, TNXA, and RP1 (see Methods). The total number of modules can be determined by three separate counts: C4A + C4B, C4L + C4S, and TNXA + 2. In all samples, these counts gave a consistent total number of modules, thus showing internal validation of results. The A33-B58-DR3 haplotype was found to be monomodular, with one long copy of C4A. The A2-B46-DR9 haplotype was found to be bimodular, with one copy of C4A and one copy of C4B, one long and one short (Table 2). We were also able to establish that APD, whose RCCX modular configuration was not previously determined, has one copy of C4A and 1 copy of C4B, both of which are long.

### Conservation of HLA-DR region between Asian and European haplotypes

**Figure 6:**
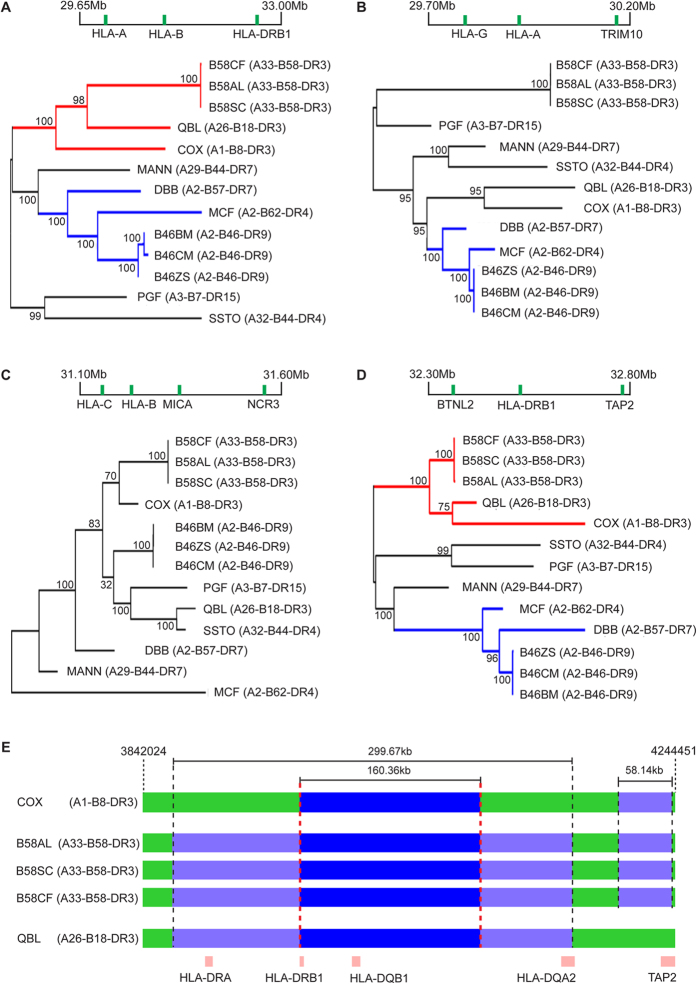
Phylogenetic relationship between Asian and European MHC haplotypes. Maximum likelihood (ML) tree derived from the SNP sequence of the MHC haplotypes covering the (**A**) chr6:29.65–33.00 Mb segment (18,781 SNPs), (**B**) chr6:29.70–30.20 Mb segment (5,111 SNPs), (**C**) chr6:31.10–31.60 Mb segment (3,617 SNPs) and (**D**) chr6:29.70–30.20 Mb segment (5,237 SNPs). The bootstrap value for each branch is indicated at the branching point. (**E**) A33-B58-DR3 SNPs comparison with COX and QBL in the HLA-DRB region. COX sequence assembly and gene annotation (HSCHR6_MHC_COX_CTG1) was used as the reference for the comparison. SNPs (4441) of B58AL, B58SC and B58CF, spanning over 402,427 bp of the COX sequence, were included in this comparison. Dark blue bars indicate shared segments found across all samples while the light blue indicates shared segments in >2 samples but not across all samples.

The availability of the Asian MHC haplotype sequences together with the eight European haplotypes offers an excellent opportunity to study the MHC haplotypic relationship and gain insights into their recent evolutionary history. Therefore, four phylogenetic trees were derived from the SNP sequences of the MHC haplotypes representing the extended MHC region (29.65–33.0 Mb), the *HLA-A* region (27.0–30.2 Mb), the *HLA-B* region (31.1–31.6 Mb), and the *HLA-DRB1* region (32.3–32.8 Mb) (Fig. 6A–D). Phylogenetic analysis indicated that the trees were typically split into two main branches and the branching was not distinguished by population differences. Indeed, the Asian haplotypes were consistently located within the same clade as specific European haplotypes, suggesting that the two Asian haplotypes did not share the most recent common ancestor. For instance, the A33-B58-DR3 haplotype cell lines were more closely related with the COX and QBL European haplotypes than with the A2-B46-DR9 haplotype over the HLA-DRB1 region. This close degree of relatedness of the Asian A33-B58-DR3 haplotype with COX and QBL is most likely because these haplotypes carry an identical HLA-DRB1*03:01 allele. Likewise, the A2-B46-DR9 haplotype shared a common ancestor with the DBB and MCF European haplotypes as a result of common possession of the HLA-A2 subtype allele. These analyses imply that the MHC haplotypic relationship is better defined by the underlying HLA allelic variation rather than by ethnicity.

The COX and QBL cell lines were previously reported to have almost identical genomic sequences across the *HLA-DRB1*, -*DQA1*, and *DQB1* genes^17^. To investigate the sequence relationship between the Asian and European DR3 haplotypes, we extracted 4441 consecutive SNPs derived from CG sequencing for the three cell lines carrying the HLA-DRB1*03:01 allele, and compared them with the COX and QBL nucleotide profiles. Given that the sequence length and gene composition are not identical between the Human Reference Sequence Assembly 37.2 and the European haplotypes at the HLA-DR region, for this analysis, the COX sequence and gene annotation was used as reference. The selected 4441 SNPs spanned over 402,427 bp of the COX sequence, encompassing the HLA-DRA, -DRB1, -DQB1, -DQA2, -DQB2, and -DOB genes. A 160-kb segment encompassing the HLA-DR genes of the A33-B58-DR3 haplotype was found to be almost identical to the COX and QBL sequence (Fig. 6E). Of the 1508 SNPs that fell within this 160-kb segment, 1506 SNPs had nucleotide profiles that matched with COX and QBL, illustrating remarkable conservation at the HLA-DR region among these haplotypes. The immediate centromeric end of this segment corresponds to a recombination hotspot, providing strong evidence for haplotype break-up. Interestingly, the conservation region range between A33-B58-DR3 cell lines and QBL was even longer, extending up to almost 300 kb. A smaller genomic segment of 58 kb containing the HLA-DOB was detected to be almost identical to COX but not QBL. Again, this 58-kb segment is flanked by recombination hotspots at both its telomeric and centromeric ends. These extreme conservations between the Asian and European haplotypes point to a shared recent common ancestor.

## Discussion

In this study, we have identified the presence of Asian conserved extended haplotypes in the A33-B58-DR3 and A2-B46-DR9 MHC haplotypes through SNP genotyping as well as deep sequencing of the extended MHC region. This provides an in-depth description of the extent and pattern of variation over several megabases within a certain CEH. In total, we have uncovered more than 200 haplotype-specific SNVs residing in each CEH, up to a third of which are not annotated in any public archives for genetic polymorphism. However, the use of the common SNP genotyping platform to interrogate the CEHs was unable to reveal the true extent of polymorphisms embedded within the conserved region. The results of this study suggest there could be additional CEH-specific private variants in other CEHs. The ability to reveal private variants that are enriched at the CEH level, but not at the population level, allows fine discrimination between individuals carrying the same CEH.

Our study demonstrated that the pattern of variations in each CEH is unique with two varieties of sequence differences. The first is the rare SNVs that spread randomly across the conserved region of the CEH; such variations have been reported in previous publications albeit at significantly smaller numbers^21^,^35^,^36^. This variation might arise through random mutations, becoming fixed in the population through subsequent expansion of the founder CEH. The second type of variation is a cluster of variations localised within the conserved region. This is evident by the hyper-variable region observed around the HLA-A region on the A33-B58-DR3 haplotype but not on the A2-B46-DR9 haplotype, and the cluster of variants found around ZFP57 in A2-B46-DR9 but not in A33-B58-DR3. Despite the apparent lack of recombination in the CEH, these concentrated spikes in variations suggest the presence of non-random mutations in the region^37^,^38^,^39^. The mechanism behind this type of variation is presumed to be the result of historic meiotic recombination where the subsequent expansions of the recombinant haplotypes give rise to different subsets of the CEH^40^. Previous studies have reported the presence of hyper-variable regions embedded in the CEHs^36^,^40^; however due to the limited resolution of SNP analysis, these studies could not comprehensively map all possible variants within hyper-variable region. In addition, these previous studies did not attempt to link SNVs and sequence differences with any direct functional role. Our study was able to perform detail variants characterization within these hyper-variable regions which was crucial in identifying variants that have a functional role. This is exemplified by the two SNVs found within the A2-B46-DR9 CEH hyper-variable region, which were shown to regulate the expression of ZFP57. Together, our results suggest that sequence differences in the CEH are likely to be due to a combination of these two types of variation instead of being dominated by one. Another interesting phenomenon is that 99% of the A33-B58-DR9 and 23% of the A2-B46-DR9 intra-CEH variants exist in the heterozygote form. This observation could well be explained by an alternate MHC evolution model known as Associative Balancing Complex (ABC)^41^. This model states that recessive detrimental mutations are built up by Muller’s ratchet effect and are sheltered by the surrounding MHC genes through LD. Moreover, these mutations exist in the heterozygote forms and, as such, natural selection is not effective in selecting against these mutations. This leads to negative epistasis and the reduction of recombination events in the MHC. The ABC evolution model supports the observation of high number of heterozygous intra-haplotypic variants in MHC CEHs and provides a possible explanation for the association of MHC CEHs with numerous diseases.

Genome-wide association studies have identified more than 100 diseases that are implicated by variants or genes within the MHC region^42^. Often, such genetic associations are not due to single, specific variants, but to the underlying MHC haplotype structure marked by extensive linkage disequilibrium^43^. These issues have complicated the identification of disease-causing variants or genes within the MHC region, especially in common CEHs, where extreme LD is often found. The identified intra-CEH variants in this study may be helpful in offering important clinical links to diseases associated with MHC CEHs, such as graft-versus-host disease (GVHD), type 1 diabetes, and nasopharyngeal carcinoma. This may allow better prediction of individuals sharing the same risk-associated CEH, and improved definition of risk-associated variants which may be enhanced within a particular CEH. Increasingly, there is evidence that non-HLA variants within the MHC region have clinical or functional associations with certain diseases^44^,^45^,^46^. Collectively, these imply that MHC-resident variants, apart from the HLA genes, could have functional implications on disease outcome. Moreover, recent studies have suggested that the exertion of *cis*-regulatory effects by variants on nearby genes, affecting the expression of target genes, is prominent within the MHC region, adding further strength to this notion^46^,^47^,^46^. Indeed, we have proven this experimentally with two A2-B46-DR9 intra-CEH variants that regulate the expression of *ZFP57* gene differentially. The presence of these MHC-residing variants offers the possibility to refine association studies for a number of MHC disease associations, which have not yet been explained, particularly the contribution of SNVs to disease phenotypes. In our study, up to 99% of intra-haplotypic variations were located within the non-coding region; these variants could be employed as a reference panel to infer the effects of non-coding variants on diseases.

There are two plausible explanations for the generation and maintenance of MHC CEHs. The first is that these long sweeps of conserved sequence haplotypes may have been driven to high frequency by positive selection over a relatively short period of time and have yet to be disrupted by recombination events^49^,^50^. A single gene or a combination of genes within the conserved stretch would be adequate to drive the haplotype expansion in the population. Another possibility is that, given the almost non-existent recombination events on haplotypes carrying a specific HLA allelic combination, these extensive conserved segments are exposed to allele-specific recombination suppression preventing haplotype breakdown^51^. The A1-B8-DR3-DQ2 haplotype has been estimated to be about 23,500 years old^21^. This relatively young age suggests that the extensive LD observed between the HLA alleles is more likely due to recent expansion, and that recombination forces have yet to act on this haplotype. Assuming that the human mutation rate per nucleotide per generation^52^ is 1.1 × 10^−8^ and a given generation is 20 years, we calculate the age of the Asian A33-B58-DR3 and A2-B46-DR9 haplotypes to be approximately 21,460 and 26,500 years old, respectively. These values are compatible with the age of the A1-B8-DR3-DQ2 haplotype, lending support to the theory that these MHC CEHs are likely to have resulted from recent expansion. In addition, it has been suggested that the expansion of CEHs is due to a single HLA allele under strong positive selection rather than epistatic selection of a specific HLA allelic combination^49^. This study reports the occurrence of sequence similarity at the HLA-DRB region between two common CEHs (A33-B58-DR3 and A1-B8-DR3-DQ2) which belong to different ethnic backgrounds but were both driven to high frequency in their respective populations. It is plausible that selection for the HLA-DRB genes drove the expansion of both of these haplotypes, and it would be interesting to identify possible environmental pressures in recent human history that may have caused such selective sweeps.

In conclusion, we demonstrated genetic fixity in two common MHC conserved extended haplotypes of Asian ancestry, A33-B58-DR3 and A2-B46-DR9, and assembled phase-resolved MHC sequences representative of these haplotypes. The availability of these Asian MHC sequences complements the eight European MHC haplotypes sequenced by the MHC Haplotype Project and provides a framework to study MHC diversity, variation, and evolution. In addition, these MHC conserved extended haplotype sequences are valuable as a resource for future disease studies on populations enriched in these haplotypes, which include many Asian populations^23^,^24^,^53^. Importantly, the discovery of intra-CEH variations, which may have functional roles, suggest that analysis of intra-CEH variation may be crucial to dissecting disease-causing genetic variants.

## Materials and Methods

### Cell lines and sequence-based HLA typing

Cells and DNA: The B-lymphoblastoid cell lines (B-LCLs), COX and QBL, were purchased from the Research Cell Bank, Fred Hutchinson Research Centre, Seattle, WA. Peripheral blood lymphocytes were isolated from healthy blood donors with informed consent. *HLA-A*, *-B*, *-C*, and *-DRB1* genes were typed by a sequence-based approach. This was achieved by interrogating the hyper-variable exons 2 and 3 of the HLA-A, -B, and -C genes by PCR amplification using specific primers, followed by direct DNA sequencing of the PCR products using ABI BigDye Terminator v3.1 chemistry run on an ABI Prism 3100 Genetic Analyser (Applied Biosystems, USA). HLA-DRB1 was sequenced and typed as described previously^54^. Purified PCR products were sequenced using the ABI BigDye Terminator v3.1 chemistry run on an ABI Prism 3100 Genetic Analyser (Applied Biosystems, USA). Excess dye terminators were removed by purification using an ethanol/EDTA/sodium acetate precipitation protocol.

### SNP genotyping, run of homozygosity analysis and SNP haplotypes analysis

Genomic DNA from the six samples (B58AL, B58SC, B58CF, B46ZS, B46BM, and B46CM) was subjected to genome-wide SNP genotyping using the Illumina Human 1 M-Duo BeadChip Kit. SNP coordinates were mapped to the Human Reference Sequence Assembly 37.2 (NCBI build 37.2), and all samples had overall SNP call rates of more than 95%. SNP loci that were not called in any of the samples or that deviated from Hardy Weinberg equilibrium at a significance level of 0.05 were not included in the downstream analysis. SNPs in chromosome 6 from 25 MB to 35 MB, covering the extended MHC region, were selected and screened for Runs of Homozygosity (ROH) segment analysis using PLINK package^55^ with the following parameters: sliding window size – 50 kb, minimum length for ROH segment –1000 kb, number of heterozygote genotype calls allowed in a window – 3, and maximum distance between adjacent SNPs in order to be considered in the segment –50 kb. In total, 10215 SNPs markers were found within the genomic region of interest and were used subsequently for the ROH analysis.

MHC panel SNPs and HLA typing data consisting of 247 individuals from the Singapore Chinese population were obtained from Lam *et al.* and the SNPs haplotype reconstruction within the MHC region was performed as described^24^.

### Identity-by-descent (IBD) analysis

B58AL, B58SC, B58CF, B46ZS, B46BM, and B46CM genotype data from the Illumina Human 1 M-Duo BeadChip from were used for IBD analysis. Independent SNPs (96,387) not in linkage disequilibrium with one another were randomly selected from the genome-wide SNPs data and were used to test for IBD among the samples. The PLINK package was used to estimate the following IBD parameters in each sample pair: probability of genetic markers sharing 0 allele (IBD = 0), probability of genetic markers sharing 1 allele IBD (IBD = 1), and probability of genetic markers sharing 2 alleles (IBD = 2).

### Genome Sequencing

Genomic DNA was extracted from B-LCLs and subjected to whole genome sequencing using next generation technology implemented by Complete Genomics (Mountain View, CA, USA). Massive parallel short paired-end sequencing was performed on self-assembling DNA nanoball arrays, generated from fragmented DNA using combinatorial probe-anchor ligation chemistry^25^. Genome assembly and variant calling protocols were carried out as previously described^56^, and the reads were aligned to the Human Reference Sequence Assembly 37.2 (NCBI build 37.2).

### MHC haplotypes variation classification and comparison

For intra-CEH comparisons, only nucleotides at positions with high quality score metrics generated from the CG assembly protocols were considered for comparison; nucleotides at positions with low confidence scores were considered ambiguous and were not used for comparison.

For inter-haplotype comparisons, consensus sequences for each of the two Asian MHC haplotypes were first established. For each haplotype, each of the three genomes would give two haploid chromosomes, and the six resulting haploid chromosomes were then compared. If two or more chromosomes had ambiguous or low confidence calls at a position, it was no-called in the consensus sequence. A variant was called if two or more haploid chromosomes showed an alternate nucleotide call.

To compare the two Asian haplotypes against the eight European MHC haplotypes sequenced by the MHC Haplotype Project, BED files of the eight European MHC haplotypes^9^,^17^,^18^ were downloaded from http://www.ucl.ac.uk/cancer/medical-genomics/mhc/#HaplotypeData and the coordinates of these BED files were aligned to the Human Reference Sequence Assembly 37.2 (NCBI build 37.2). Genetic variants found between the MHC haplotypes were annotated using the ANNOVAR software^57^. Construction of the consensus sequences as well as the intra and inter-haplotype comparisons were performed using in-house generated R-scripts.

### Assessment of sequencing accuracy

Primer pairs to assess the variants were designed using NCBI Primer-BLAST and checked against the Human Reference Sequence Assembly 37.2 (NCBI build 37.2). Only primer pair sequences matching the Asian MHC haplotypes were selected. The sequences of these primer pairs and the position of variants of interest can be found in Supplementary Table S4-S5 and Table S8. PCR amplification was performed and the PCR products were sequenced.

The PCR template generated from primer sequences AGCAGTCACAAGTCACAGGG and CAGCCCATCGCATGCTCAAT was selected and subjected to TA-cloning. TA-cloning was then performed using pGEM®-T Easy Vector Systems (Promega, Fitchburg, WI), according to the manufacturer’s instructions. Colonies were selected and plasmid DNA was extracted using the QIAprep® Miniprep Kit (Qiagen), and sequenced with the T7 promoter and SP6 promoter primers.

### qRT-PCR for ZFP57 expression quantification

Cultures at 1 × 10^6^ cells/ml were treated with 200 nM phorbol 12-myristate 13 acetate (PMA, Sigma) and 125 nM ionomycin for 6 h. Total RNA was extracted using the RNeasy Mini Kit (Qiagen), from which cDNA was generated using the Maxima® First Strand cDNA Synthesis Kit (Thermo Fisher Scientific). qPCR by KAPA SYBR® FAST Roche LightCycler® 480 2X qPCR Master Mix (Kapa Biosystems, Woburn, MA) was performed in triplicate for each of the two biological replicates on the Roche LightCycler® 480 System (Roche Applied Science). Ct value calculations using the second derivative maximum method and melting curve analysis were carried out with gene-specific primer pairs. *ZFP57* expression was normalized to Hypoxanthine Phosphoribosyltransferase 1 (*HPRT1*) and determined using the ΔCt method. Primer pair sequences were as follows: ZFP57 forward - TGAGGATGTGGCAGTGAATTT, ZFP57 reverse - GTGTTTGGGAGATGGACAAAC, HPRT1 forward - GTAATGACCAGTCAACAGGGGAC, HPRT1 reverse - CCAGCAAGCTTGCGACCTTGACCA.

### Phylogenetic analysis

The SNP sequences of the Asian and European haplotypes were comprised of 18,781 common SNPs annotated in dbSNP build 137. SNP positions with heterozygous calls in the Asian haplotypes were denoted as missing data. Phylogenetic trees were constructed based on the maximum likelihood statistical method, and the Kimura 2-parameter substitution model was used to calculate the likelihood on a given tree. To evaluate the reliability of branching points, a bootstrap test of phylogeny was performed (n = 500). The tree building process was implemented in MEGA5^58^.

### Linkage disequilibrium and haplotype analysis in chr6:29.60–29.69(Mb)

The LD structure in this region was established based on the genotype information of 163 intra-CEH variants in 105 unrelated Southern Han Chinese individuals (1000 Genome Project)^30^,^31^. r^2^ was used as a measurement of LD between variants, and an LD block was defined as a region with an SNP pairwise r^2^ value >0.8. The computation of r^2^ and the haplotype analysis within each LD block were performed using Haploview^59^.

### Luciferase assay

To evaluate the potential regulatory role of intra-CEH variants in *ZFP57* transcription, a luciferase reporter assay was designed to test the effect of 41-bp DNA sequences bearing the different allele of each intra-CEH variant flanked by spanning sequences of 20 bp. The 41-bp DNA fragments were annealed and directly cloned between Sac I and Xho I restriction sites upstream of the minimal SV40 promoter in the pGL3-Promoter vector (Promega). Annealing primers are listed in Supplementary Table S12. For the luciferase assay, each promoter reporter construct (4 mg/ml) was co-transfected with 40 ng/ml of Renilla control vector into HT1080 cells seeded in 96-well microtiter plates. Luciferase activity was measured 30 h post transfection by the Dual-Luciferase Reporter Assay System (Promega). The normalized luciferase activity of each construct (Firefly/Renilla ratio) was compared with that of the pGL3-promoter control-transfected cells.

### Elucidation of RCCX copy number variations (CNV) in the cell lines

Genomic DNA was extracted from the six cultured cell lines (B58AL, B58SC, B58CF, B46ZS, B46BM, and B46CM) using the QIAGEN® DNeasy Blood and Tissue Kit, according to the manufacturer’s protocol. The assay used to determine the RCCX modular duplication in each cell line was developed from a modified version of the real-time PCR assay, as previously described^60^. Primers specific for C4A, C4B, C4L, and C4S were designed to resolve the RCCX modular duplication (Supplementary Table S13). Amplicons for C4L and C4S shared a common forward primer, and their reverse primers were designed to differentiate between the long and short C4 gene. In addition, the copy number of the TNXA gene, which equals the number of RCCX modules minus two, was also interrogated. The assignment for the number of copies of each targeted gene involved two calibration steps. The first calibration step was a quantitative real-time PCR endogenous control using the RP1 gene, which is positioned upstream of the RCCX module and always present as one copy per chromosome. Quantified levels of target genes were compared to levels of RP1 in order to obtain relative copy numbers of target genes. However, in this approach, there is an intrinsic underestimation of the targeted gene copy number. To correct for this underestimation, a second calibration step was performed. A calibrated plot created from 13 reference human cell lines (COX, QBL, MOU, PGF, SSTO, DBB, WT51, MADURA, CB6B, WT8, DAUDI, MANIKA, and HOM) with known RCCX modular numbers were used to assign the targeted gene copy number unambiguously. The genomic DNAs of these reference cell lines were purchased from the Research Cell Bank, Fred Hutchinson Research Centre, Seattle, WA. For each sample, the number of copies of C4A + C4B, C4L + C4S, and TNXA + 2 were the same, acting as an internal validation.

## Additional Information

**Accession codes**: Data reported in this study will be available at NCBI SRA (Accession ID: SRP053276).

**How to cite this article**: Lam, T.H. *et al.* Intrahaplotypic Variants Differentiate Complex Linkage Disequilibrium within Human MHC Haplotypes. *Sci. Rep.* **5**, 16972; doi: 10.1038/srep16972 (2015).

## Supplementary Material

Supplementary Information

## Figures and Tables

**Table 1:** Intra- and inter-haplotypic comparisons: SNVs and indels.

Variant Type	Intra-CEH				Inter-haplotype			
	A2-B46-DR9	A33-B58-DR3	A2-B46-DR9 vs A33-B58-DR3	A2-B46-DR9 vs PGF	A33-B58-DR3 vs PGF	A2-B46-DR9 vs COX	A33-B58-DR3 vs COX	PGF vs COX
**SNVs**								
Coding	2	1	161	126	204	160	206	190
Missense	1	1	84	68	111	91	113	99
Nonsense	0	0	1	2	1	0	1	2
Synonymous	1	0	76	56	92	69	92	89
ncRNA exonic	0	0	138	106	119	148	152	115
UTR 5’	1	1	24	28	34	24	32	37
UTR 3’	0	11	75	54	87	64	79	78
Intronic	32	38	1745	1320	1704	1577	1859	1529
Promoter region	6	5	217	228	242	267	222	195
Intergenic	197	234	5141	4250	6795	4779	6549	7911
**Total**	**238**	**290**	**7501**	**6112**	**9185**	**7019**	**9099**	**10055**
**SNVs/100kb**	**8.58**	**6.84**	**270**	**220**	**211**	**253**	**217**	**237**
								
**Indels**								
Coding	0	0	3	2	2	5	4	3
Frameshift	0	0	3	2	2	3	3	1
Non-frameshift	0	0	0	0	0	2	1	2
ncRNA exonic	1	0	17	9	18	18	24	19
UTR	1	1	12	6	18	9	16	15
Intronic	9	12	208	135	180	298	363	381
Promoter region	3	2	26	19	19	37	44	36
Intergenic	37	37	418	298	516	688	993	1298
**Total**	**51**	**52**	**684**	**469**	**753**	**1055**	**1444**	**1752**
**Indels/100kb**	**1.80**	**1.22**	**24.6**	**16.9**	**17.3**	**37.9**	**34.4**	**41.2**

BED files of the PGF and COX 9,17,18 were downloaded from http://www.ucl.ac.uk/cancer/medical-genomics/mhc/#HaplotypeData and the coordinates of these BED files were aligned to the Human Reference Sequence Assembly 37.2 (NCBI build 37.2).

**Table 2:** RCCX modular structure in the six Asian cell lines.

RCCX Structure	Sample	C4A	C4B	C4L	C4S	TNXA	
Monomodular	B58SC	2	0	2	0	0	
	B58AL	2	0	2	0	0	
	B58CF	2	0	2	0	0	
Bimodular	B46BM	2	2	2	2	2	
	B46ZS	2	2	2	2	2	
	B46CM	2	2	2	2	2	
	APD	2	2	4	0	2	
