# Supplementary material for: Intrahaplotypic Variants Differentiate Complex Linkage Disequilibrium within Human MHC Haplotypes
Source: Sci Rep. 2015 Nov 23;5:16972. doi: 10.1038/srep16972 (PMC4655331; doi:10.1038/srep16972)
Supplement: Supplementary Information [file srep16972-s1.pdf]

Intrahaplotypic Variants Differentiate Complex Linkage Disequilibrium within Human MHC Haplotypes

Tze Hau Lam[1, 2] Matthew Zirui Tay[1, 3], Bei Wang[1], Ziwei Xiao[1], Ee Chee Ren[1,2*]

Supplementary Figure Legends:

Figure S1. SNP alignment of independent A33-B58-DR3 and A2-B46-DR9 chromosomes with consensus SNPs sequence of each haplotype. Green columns indicate SNP alleles identical to the consensus sequence while dark green columns indicate otherwise.

Figure S2. Sequencing coverage and no-call rate across chr6:25.0 – 35.0(Mb). No-call and coverage profile for each cell line across the MHC region. The average GC-corrected coverage and the no-call counts were binned into non-overlapping 20-kb windows.

Figure S3. Zygosity profile of variants derived from CG sequencing. The region chr6: 25Mb–35Mb was binned into non-overlapping windows of 20 kb. The number of homozygous and heterozygous variants with respect to the NCBI Build 37.2 reference sequence was calculated in each bin. For each of the six individuals, the upper panel plot represents the homozygous variants counts while the lower panel plot represents the heterozygous variants counts across the region of interest.

Figure S4. Spikes in intra-CEH variations in the **(A)** A2-B46-DR9 haplotype and **(B)** A33-B58-DR3 haplotype. The variation counts were binned into non-overlapping 2-kb windows. Heightened intra-CEH variation in genomic segments extended to 120 kb and 240 kb were identified in the A2-B46-DR9 and A33-B58-DR3 haplotype, respectively.

Figure S5. Alignment of B58AL DNA templates derived via TA cloning. The DNA template was mapped to NCBI Build 37.2 reference sequence chr6:29,912,732–29,913,130 located within the HLA-A gene. The derived DNA sequences were also aligned with the sequence of the A*33:03 allele downloaded from IMGT/HLA database (http://www.ebi.ac.uk/ipd/imgt/hla/, Release 3.13.1).

Figure S6. Alignment of B58SC DNA templates derived via TA cloning. The DNA template was mapped to NCBI Build 37.2 reference sequence chr6:29,912,732–29,913,130 located within the HLA-A gene. The derived DNA sequences were also aligned with the sequence of the A*33:03 allele downloaded from IMGT/HLA database (http://www.ebi.ac.uk/ipd/imgt/hla/, Release 3.13.1).

Figure S7. Alignment of B58CF DNA templates derived via TA cloning. The DNA template was mapped to NCBI Build 37.2 reference sequence chr6:29,912,732–29,913,130 located within the HLA-A gene. The derived DNA sequences were also aligned with the sequence of the A*33:03 allele downloaded from IMGT/HLA database (http://www.ebi.ac.uk/ipd/imgt/hla/, Release 3.13.1).

Figure S8. Variant counts between pairwise sequence comparisons of B46BM, B46CM, and B46ZS with the A33-B58-DR3 haploid sequence. A sliding window of 1 kb in length was used to analyse nucleotide differences between the sequences in the MHC region of chr6:29.60–29.69(Mb).

Figure S9. Intra-CEH variants overlapping with H3K4me1 peaks and their respective allele-specific luciferase activity. **(A)** H3K4me1 levels at the genomic location of the intra-CEH variants. The H3K4me1 level at these sites are referenced from the B-LCL cell line GM12878 from the ENCODE data. **(B)** Luciferase activity for each allele of intra-CEH variants. Reporter plasmids carrying the fragment sequence of each unique allele of a certain variant were co-transfected with Renilla control vector into HT1080 cells. The luciferase activity readout was normalized to Renilla luciferase, and the representative results from three independent experiments are shown.

Figure S1

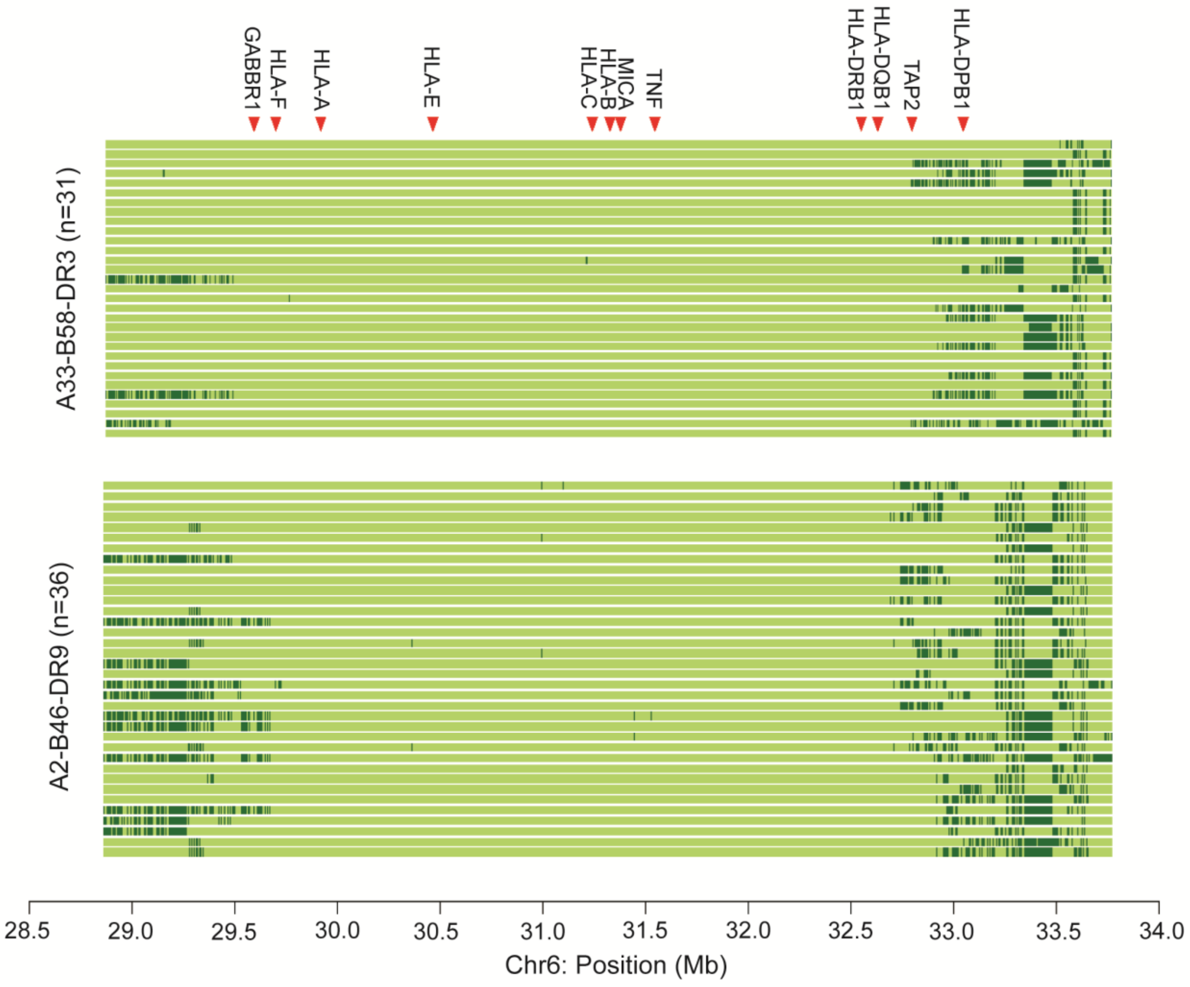
GABBR1
HLA-F
HLA-A
HLA-E
HLA-C
HLA-B
MICA
TNF
HLA-DRB1
HLA-DQB1
TAP2
HLA-DPB1
A33-B58-DR3 (n=31)
A2-B46-DR9 (n=36)
28.5
29.0
29.5
30.0
30.5
31.0
31.5
32.0
32.5
33.0
33.5
34.0
Chr6: Position (Mb)

Figure S2

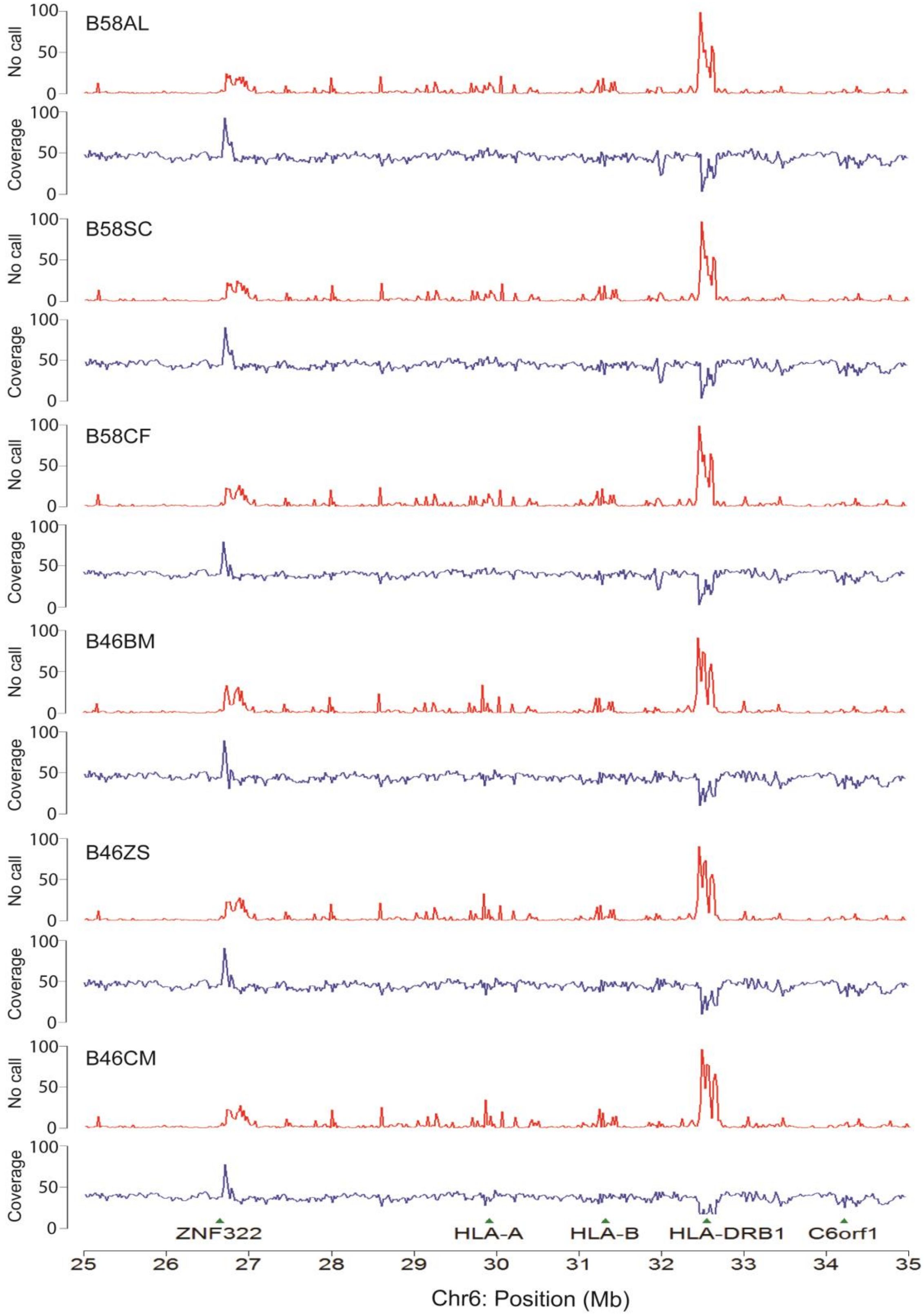

B58AL
B58SC
B58CF
B46BM
B46ZS
B46CM
No call
Coverage
100
50
0
ZNF322
HLA-A
HLA-B
HLA-DRB1
C6orf1
25
26
27
28
29
30
31
32
33
34
35
Chr6: Position (Mb)

Figure S3

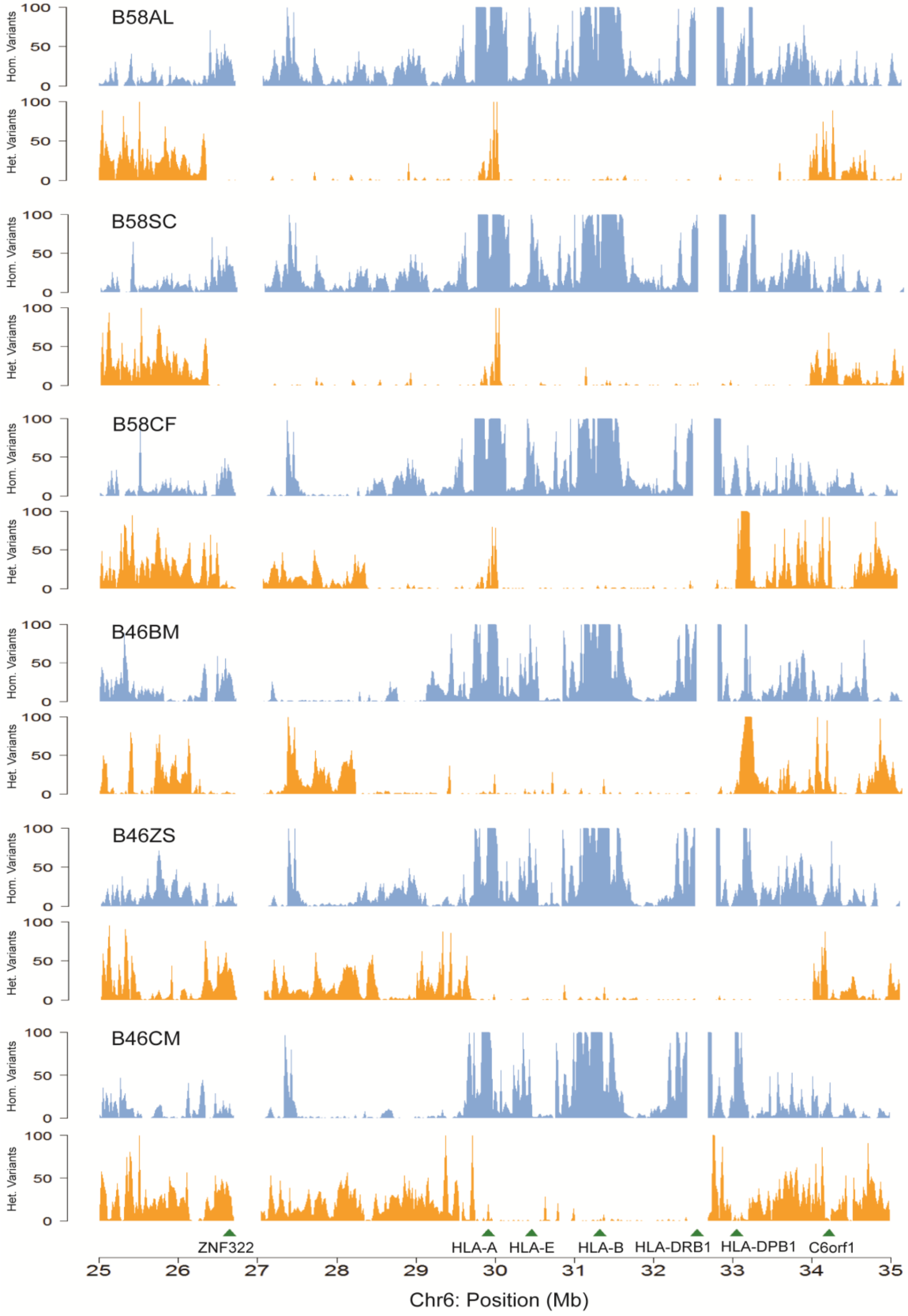
B58AL
B58SC
B58CF
B46BM
B46ZS
B46CM
Hom. Variants
Het. Variants
100
50
0
ZNF322
HLA-A
HLA-E
HLA-B
HLA-DRB1
HLA-DPB1
C6orf1
25
26
27
28
29
30
31
32
33
34
35
Chr6: Position (Mb)

Figure S4

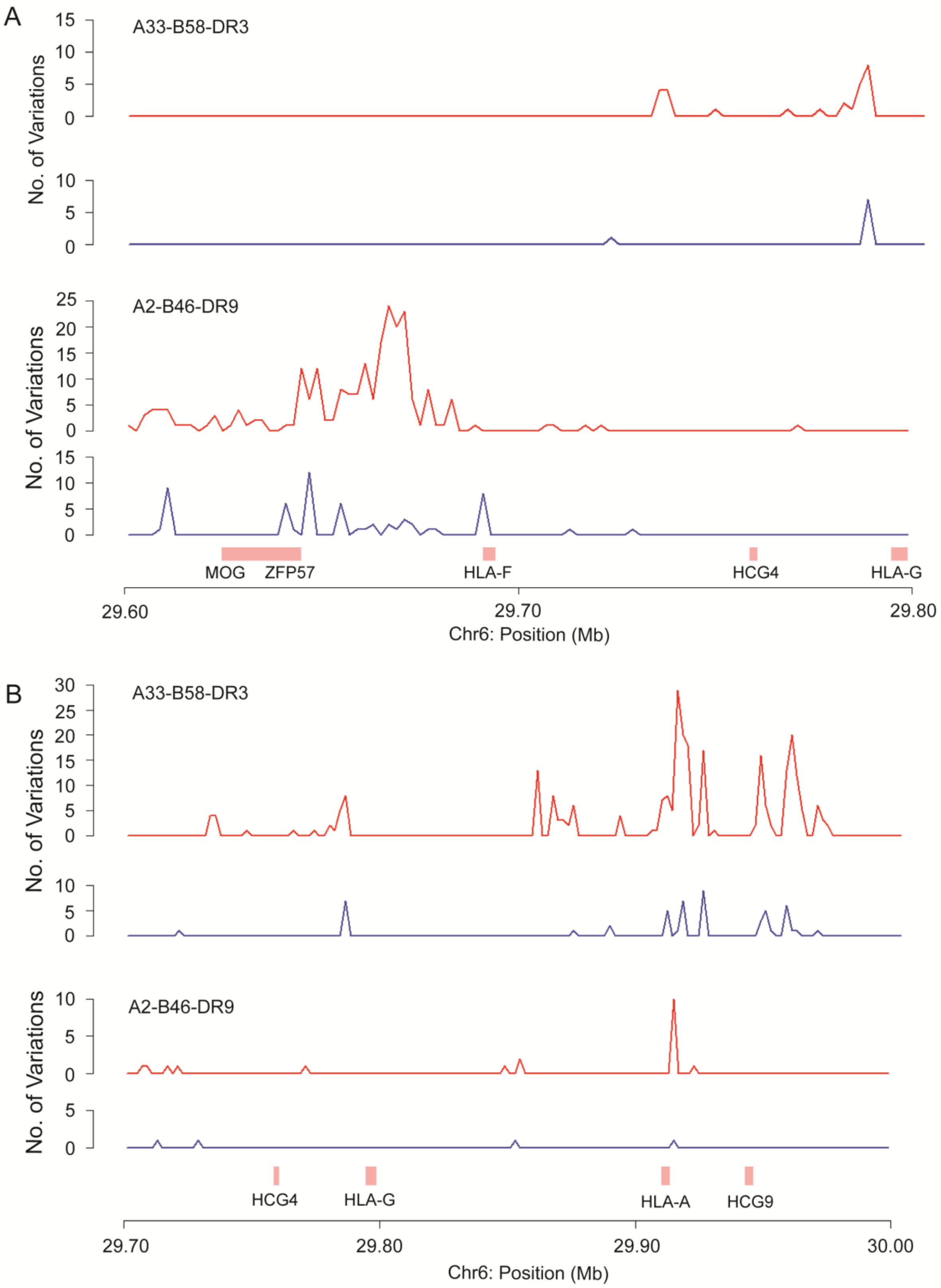

A
A33-B58-DR3
No. of Variations
15
10
5
0
10
5
0
A2-B46-DR9
No. of Variations
25
20
15
10
5
0
15
10
5
0
MOG
ZFP57
HLA-F
HCG4
HLA-G
29.60
29.70
29.80
Chr6: Position (Mb)
B
A33-B58-DR3
No. of Variations
30
25
20
15
10
5
0
10
5
0
A2-B46-DR9
No. of Variations
10
5
0
5
0
HCG4
HLA-G
HLA-A
HCG9
29.70
29.80
29.90
30.00
Chr6: Position (Mb)

Figure S5

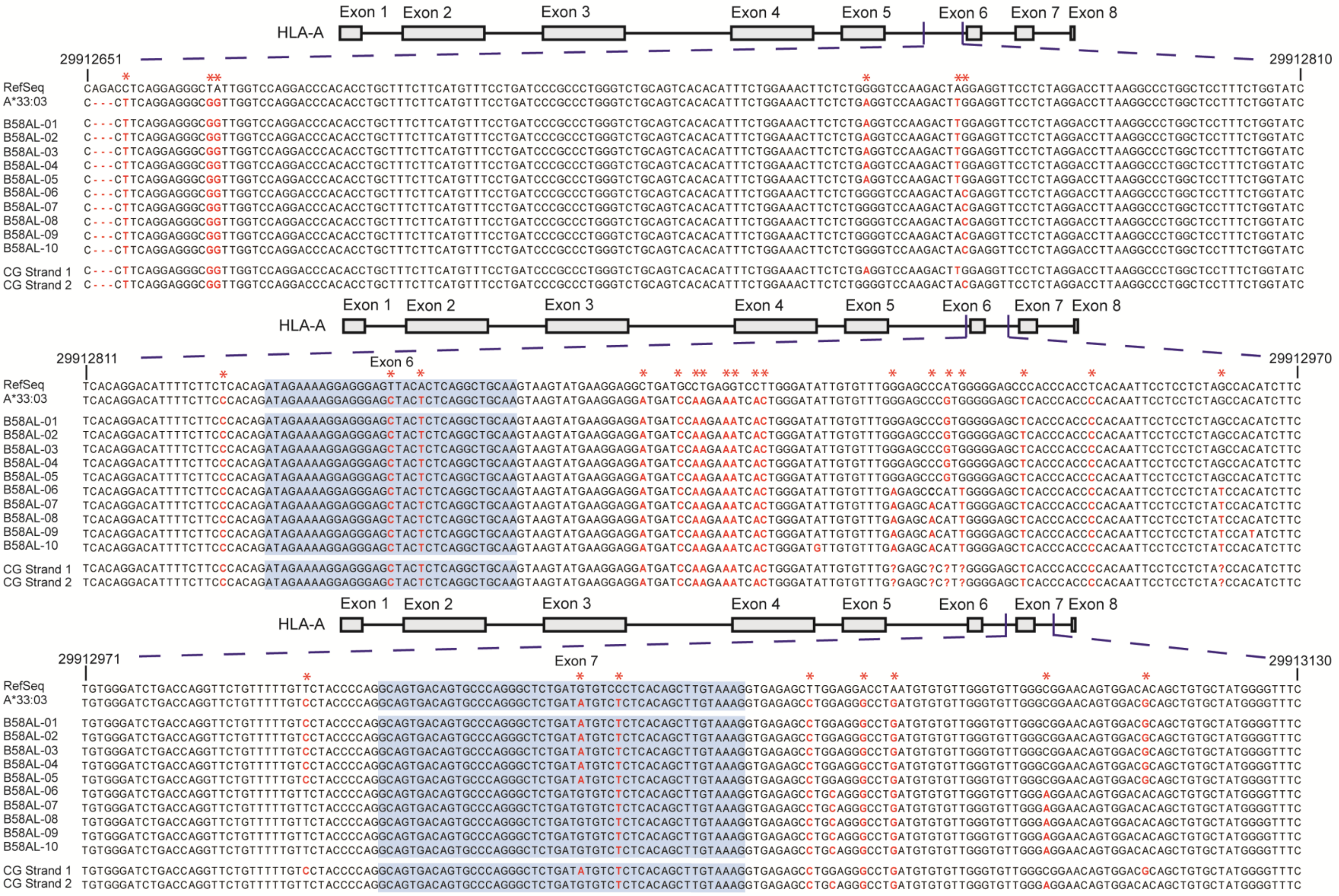
HLA-A
Exon 1
Exon 2
Exon 3
Exon 4
Exon 5
Exon 6
Exon 7
Exon 8
29912651
29912810
29912811
29912970
29912971
29913130
RefSeq
A*33:03
B58AL-01
B58AL-02
B58AL-03
B58AL-04
B58AL-05
B58AL-06
B58AL-07
B58AL-08
B58AL-09
B58AL-10
CG Strand 1
CG Strand 2

Figure S6

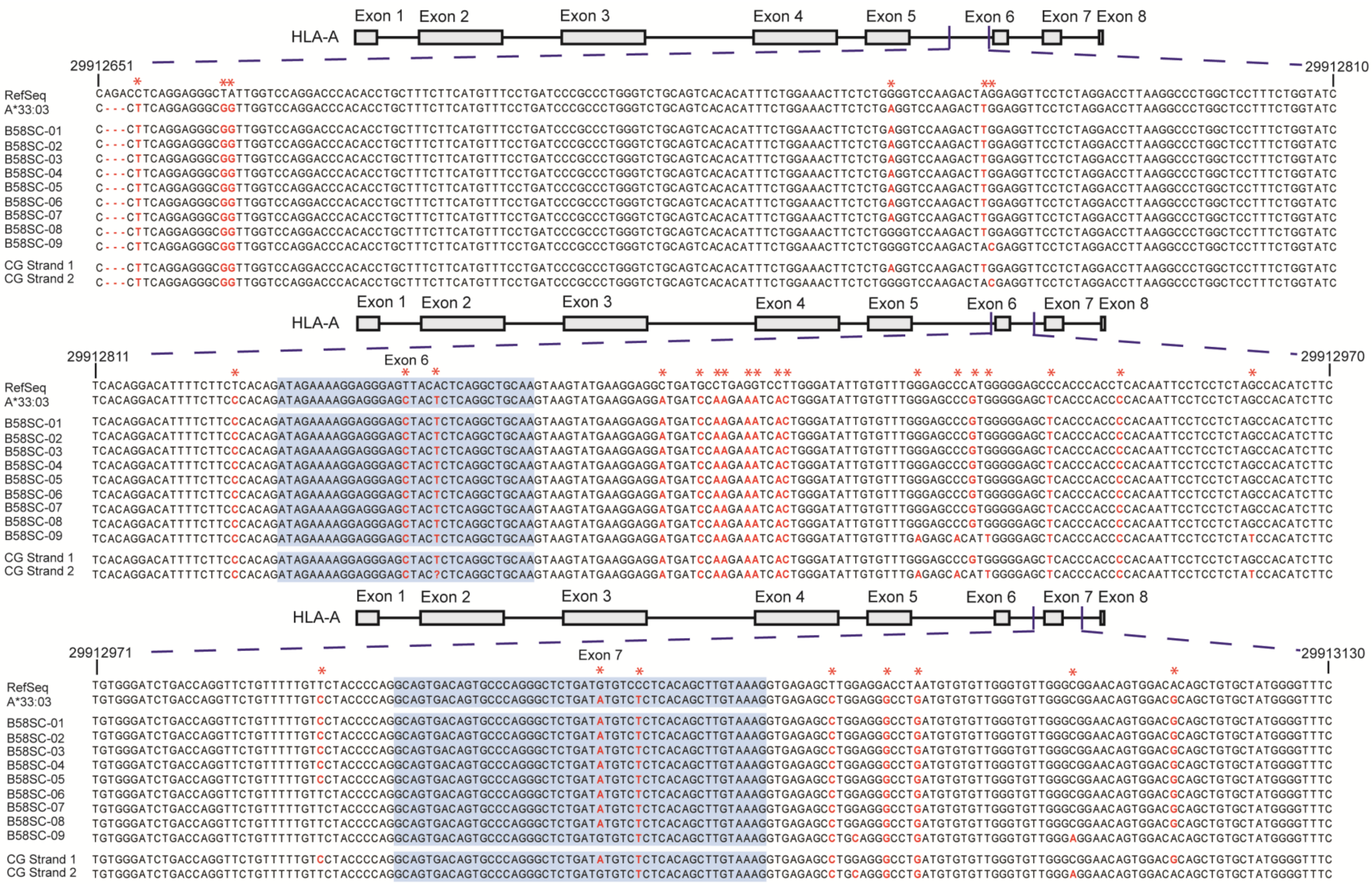

HLA-A
Exon 1
Exon 2
Exon 3
Exon 4
Exon 5
Exon 6
Exon 7
Exon 8
29912651
29912810
29912811
29912970
29912971
29913130
RefSeq
A*33:03
B58SC-01
B58SC-02
B58SC-03
B58SC-04
B58SC-05
B58SC-06
B58SC-07
B58SC-08
B58SC-09
CG Strand 1
CG Strand 2

Figure S7

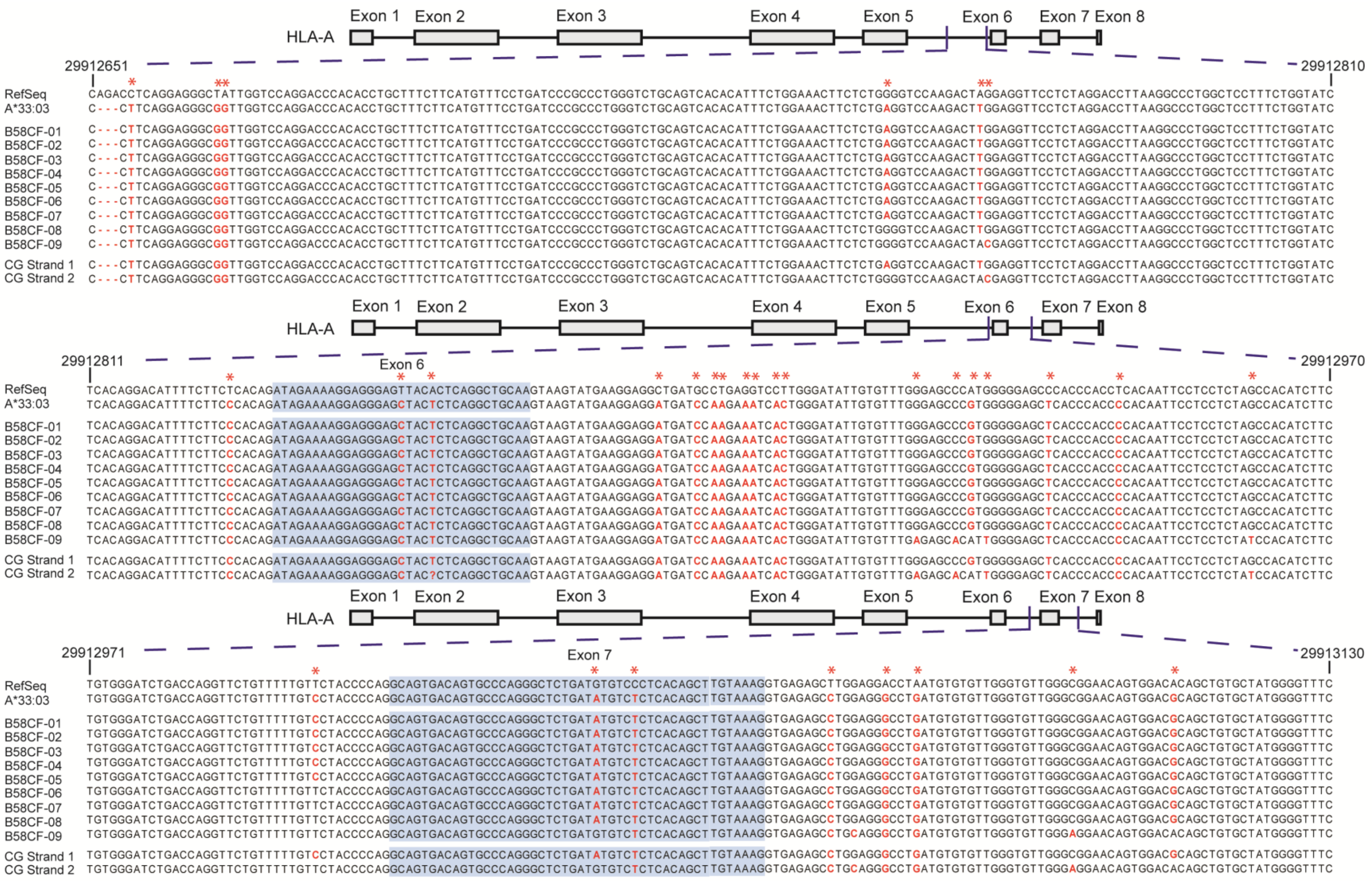

Figure S8

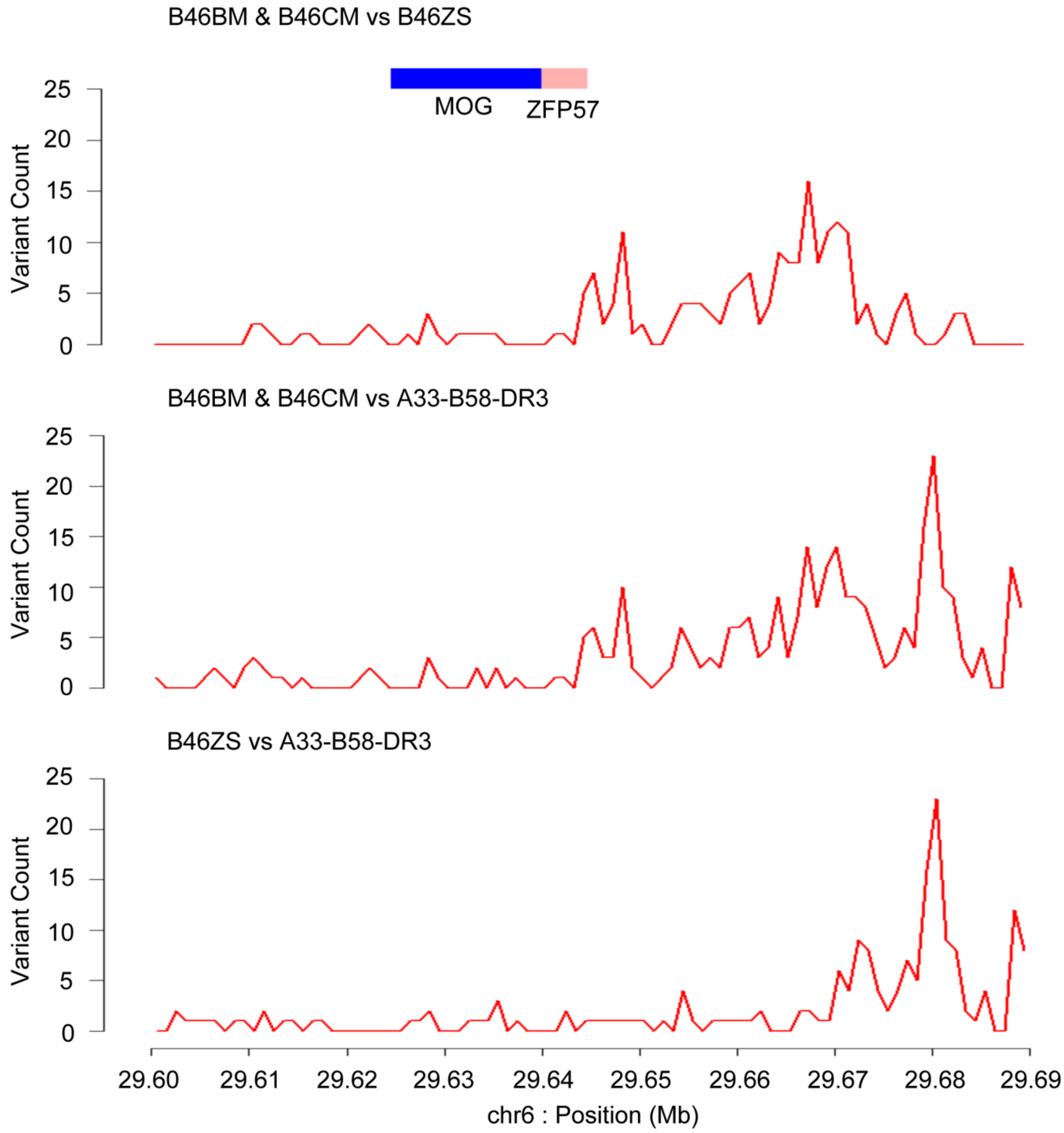

B46BM & B46CM vs B46ZS
MOG
ZFP57
Variant Count
25
20
15
10
5
0
B46BM & B46CM vs A33-B58-DR3
Variant Count
B46ZS vs A33-B58-DR3
Variant Count
29.60
29.61
29.62
29.63
29.64
29.65
29.66
29.67
29.68
29.69
chr6 : Position (Mb)

Figure S9

A

rsID	Position	H3k4me1 Signal
rs3117318	29611781	4.52
rs3129073	29615822	1.92
rs3117295	29653377	4.08
rs7738158	29667527	3.48
rs3129056	29670250	3.6
rs3129048	29670555	3.16
rs3129047	29670572	3.16
rs1610588	29676715	11.32
rs1610589	29677386	17.36

B

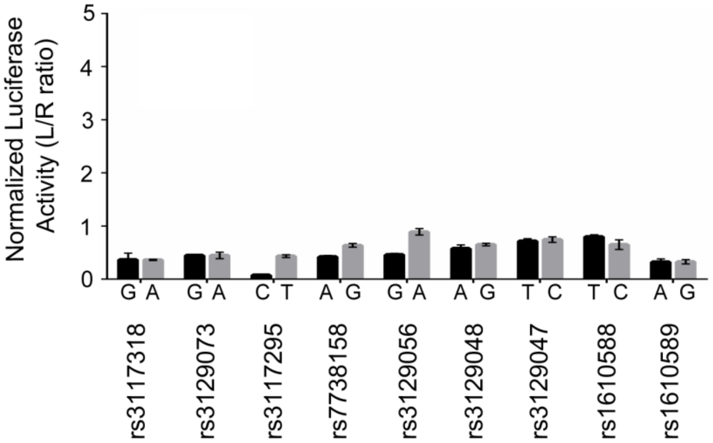

Table S1. Range of conserved extended region within the MHC for the six cell lines.

	SNP Genotyping Platform					CG Sequencing Platform			
Sample ID	Start	End	Length (Mb)	No. of SNPs	No. of het SNPs	Start	End	Length (Mb)	No. of het variants
B58AL	26922906	33853071	6.93	8660	26	26350000	33830000	7.48	595
B58SC	26922906	33820059	6.90	8637	26	26350000	33810000	7.46	578
B58CF	28310997	32973794	4.66	6997	35	28350000	32950000	4.60	455
B46BM	29350854	32903900	3.57	6238	25	29367500	32917500	3.55	221
B46ZS	29577617	33910884	4.33	6975	21	29630000	33890000	4.26	146
B46CM	29728209	32739888	3.01	5283	16	29550000	32650000	3.10	167

Table S2. CG sequencing coverage and performance.

Cell lines	Mean Coverage Per bp	% bp Coverage >5X	% bp Coverage >20X	% bp Coverage >40X	% of MHC region covered
B58AL	44.67	98.14	91.09	61.40	95.31
B58SC	43.69	98.10	90.57	59.44	95.15
B58CF	39.06	97.96	88.14	49.10	94.66
B46BM	43.54	98.13	90.31	58.93	95.03
B46ZS	44.30	98.11	90.88	60.94	95.06
B46CM	37.13	97.86	86.64	44.20	94.18

Table S3. SNP genotype calls differences between Illumina Human 1M-Duo BeadChip and CG sequencing. Hmz indicates homozygous genotype call and htz indicates heterozygous genotype call.

Sample ID	Positions Compared	Differences hmz (1M-Duo) /hmz (CG)	Differences hmz (1M-Duo) /htz (CG)	Differences htz (1M-Duo/ hmz (CG)	Differences Total	% Match
B58AL	9916	15	1	23	39	99.6
B58SC	9876	14	4	26	44	99.6
B58CF	9880	12	8	27	47	99.5
B46BM	9908	11	8	14	33	99.7
B46ZS	9903	13	3	14	30	99.7
B46CM	9904	12	10	13	35	99.6

Table S4. Re-sequencing experiments to assess mismatches between SNP genotyping and CG sequencing platform in samples carrying A33-B58-DR3.

					B58AL			B58SC			B58CF		
Forward Primer	Reverse Primer	Position	rsID	Ref	SNP Array	CG	PCR	SNP Array	CG	PCR	SNP Array	CG	PCR
ACTGACAGAATGAAC CTGCAGAC	AATCACTCTCTGGTA CAGGATCTGG	29,796,376	rs12722477	C	C/C	A/A	?/?	C/C	A/A	A/A	C/C	A/A	A/A
TGAGAACTGGCGGG GAGATA	TCTCTTGCTGGCTCA GCTTT	29,819,909	rs2508053	C	C/T	C/C	C/C	C/T	C/C	C/C	C/T	C/C	C/C
CTGACTCATATCAAG GGCCAGAAA	AGAGAGGAAAGTCA GGACACAATAC	30,383,046	GA005234	C	C/G	C/C	C/C	C/G	C/C	C/C	C/G	C/C	C/C
ATAAAAACAGGCTGC ATGTGGTAAA	AGTTGAGGTTTTTCT GTTATGCCTG	30,418,354	rs34111681	G	T/T	G/G	G/G	T/T	G/G	G/G	T/T	G/G	G/G
CAGAACCAGGGAGA TGAGACATAC	TGTTCCTGCTTCTCT TTTCACTTTC	31,170,514	rs9263870	A	A/G	G/G	G/G	A/G	G/G	G/G	A/G	G/G	G/G
GAACATATGCTACAA AAGGCCAGAG	GGTGTGGAGAAGGC TGTGGG	31,321,327	rs9266095	A	A/G	A/A	A/A	A/G	A/A	A/A	T/T	G/G	?/?
CTCTTGAAGGACTCT GGGTTAGAAG	GCACCAGAGTTCAA GAGAGAAAATTA	31,639,979	rs9267532	C	C/T	T/T	T/T	C/T	T/T	T/T	C/T	T/T	T/T
CAATGCTTATAGGGT ATCCCCAGTC	GCAGTGTACACACA CAGATACTGAT	31,655,438	rs10573	G	A/G	A/A	A/A	A/G	A/A	A/A	A/G	A/A	A/A
TAGGGTCTCTAATCT CCAAAACACC	CTAAAAGCCAGAGC TCCCAGTCC	31,697,558	rs707916	G	A/G	A/A	?/?	A/G	A/A	?/?	A/G	A/A	A/A
CCTTTATGAGACCTG CATTGAACC	GGTACTCCAACACTG ATCATAGGG	32,130,937	rs10680	T	C/C	T/T	T/T	C/C	T/T	T/T	C/C	T/T	?/?
TCAGATTGAATTTTT CCTCCCTTCC	GATTACAGCTTCCAC AAGTTCCATT	33,036,549	rs17509489	T	G/G	T/T	?/?	G/G	T/T	T/T	G/G	T/T	?/?

Table S5. Re-sequencing experiments to assess mismatches between SNP genotyping and CG sequencing platform in samples carrying A2-B46-DR9.

					B46BM			B46ZS			B46CM		
Forward Primer	Reverse Primer	Position	rsID	Ref	SNP Array	CG	PCR	SNP Array	CG	PCR	SNP Array	CG	PCR
AGTACATGTAGACAG CTCACAGT	GCACAGGGAATGTG TTCTCG	29,801,958	rs2743944	T	T/C	T/T	T/C	T/C	T/T	T/C	T/C	T/T	T/C
GGGGTTTCTTTGCAT TGGATGTATT	TTGTCTCTTGATACC ACAAGGAGAT	29,913,509	rs1062405	T	C/C	T/T	T/T	C/C	T/T	T/T	C/C	T/T	T/T
ATAGAATTAGAAAGA GGCTGGGGTC	GTGCTAATGAAAGTT GGGCCTTAG	29,942,191	rs2232236	T	G/G	del/del	del/del	G/G	del/del	?/?	G/G	del/del	del/del
CTTTCAGTTCTCTTC TGTGTCTCCA	AGTATATTAGGTTAG CGGGTGGTAG	30,704,985	rs28380598	T	C/C	T/T	T/T	C/C	T/T	T/T	C/C	T/T	T/T
CCGTGGGGATGGCT AGAAAA	CCCTGAGGGAATCT GGGGTA	31,079,236	rs1265055	G	A/G	A/A	A/A	A/G	A/A	A/A	A/G	A/A	A/A
GATTCCAGACTTGGA GTTTCAACAG	GAGTAAAGGACTGA GAGGATGGGA	31,082,304	rs3130554	T	G/T	T/T	T/T	G/T	T/T	T/T	G/T	T/T	T/T
CCTTTATGAGACCTG CATTGAACC	GGTACTCCAACACTG ATCATAGGG	32,130,937	rs10680	T	C/C	T/T	T/T	C/C	T/T	T/T	C/C	T/T	?/?
TCAGATTGAATTTTT CCTCCCTTCC	GATTACAGCTTCCAC AAGTTCCATT	33,036,549	rs17509489	T	G/G	C/T	C/T	G/G	T/T	T/T	G/G	T/T	T/T

Table S6. Intra-CEH SNVs within coding sequence region.

A2-B46-DR9

Position Start	Position End	Alternate Allele	Reference Allele	Variant Type	Effect	Gene	Exon
29,634,002	29,634,003	C	G	SNV	missense	MOG	3
32,190,483	32,190,484	A	G	SNV	synon	NOTCH4	3

A33-B58-DR3

Position Start	Position End	Alternate Allele	Reference Allele	Variant Type	Effect	Gene	Exon
29,913,036	29,913,037	A	G	SNV	missense	HLA-A	7

Table S7. Variants within DNA template chr6:29,912,732 – 29,913,130.

Position Start	Position End	Region	VarType	Zygosity	Reference	B58AL		B58SC		B58CF	
						CG Sequence	TA Cloning	CG Sequence	TA Cloning	CG Sequence	TA Cloning
29,912,652	29,912,657	Intron	SUB	Hom	AGACC	CT/CT	CT/CT	CT/CT	CT/CT	CT/CT	CT/CT
29,912,667	29,912,669	Intron	SUB	Hom	TA	GG/GG	GG/GG	GG/GG	GG/GG	GG/GG	GG/GG
29,912,753	29,912,754	Intron	SNV	Het	G	G/A	G/A	G/A	G/A	G/A	G/A
29,912,765	29,912,766	Intron	SNV	Het	A	A/T	A/T	A/T	A/T	A/T	A/T
29,912,766	29,912,767	Intron	SNV	Het	G	G/C	G/C	G/C	G/C	G/C	G/C
29,912,829	29,912,830	Intron	SNV	Hom	T	C/C	C/C	C/C	C/C	?/?	C/C
29,912,851	29,912,852	Exon 6	SNV	Hom	T	C/C	C/C	C/C	C/C	C/C	C/C
29,912,855	29,912,856	Exon 6	SNV	Hom	A	T/T	T/T	T/?	T/T	T/T	T/T
29,912,884	29,912,885	Intron	SNV	Hom	C	A/A	A/A	A/A	A/A	A/A	A/A
29,912,889	29,912,893	Intron	SUB	Hom	GCCT	CCAA/CCAA	CCAA/CCAA	CCAA/CCAA	CCAA/CCAA	CCAA/CCAA	CCAA/CCAA
29,912,895	29,912,897	Intron	SUB	Hom	GG	AA/AA	AA/AA	AA/AA	AA/AA	AA/AA	AA/AA
29,912,899	29,912,901	Intron	SUB	Hom	CT	AC/AC	AC/AC	AC/AC	AC/AC	AC/AC	AC/AC
29,912,917	29,912,918	Intron	SNV	Het	G	?/?	G/A	G/A	G/A	?/?	G/A
29,912,922	29,912,923	Intron	SNV	Het	C	?/?	C/A	C/A	C/A	?/?	C/A
29,912,924	29,912,925	Intron	SNV	Het	A	?/?	A/G	A/G	A/G	?/?	A/G
29,912,926	29,912,927	Intron	SNV	Het	G	?/?	G/T	G/T	G/T	?/?	G/T
29,912,934	29,912,935	Intron	SNV	Hom	C	T/T	T/T	T/T	T/T	T/T	T/T
29,912,943	29,912,944	Intron	SNV	Hom	T	C/C	C/C	C/C	C/C	C/C	C/C
29,912,960	29,912,961	Intron	SNV	Het	G	?/?	G/T	G/T	G/T	G/?	G/T
29,913,000	29,913,001	Intron	SNV	Het	T	T/C	T/C	T/C	T/C	T/C	T/C
29,913,036	29,913,037	Exon 7	SNV	Het	G	G/A	G/A	G/A	G/A	G/A	G/A
29,913,041	29,913,042	Exon 7	SNV	Hom	C	T/T	T/T	T/T	T/T	T/T	T/T
29,913,066	29,913,067	Intron	SNV	Hom	T	C/C	C/C	C/C	C/C	C/C	C/C
29,913,069	29,913,070	Intron	SNV	Het	G	G/C	G/C	G/C	G/C	G/C	G/C
29,913,073	29,913,074	Intron	SNV	Hom	A	G/G	G/G	G/G	G/G	G/G	G/G
29,913,077	29,913,078	Intron	SNV	Hom	A	G/G	G/G	G/G	G/G	G/G	G/G
29,913,097	29,913,098	Intron	SNV	Het	C	C/A	C/A	C/A	C/A	C/A	C/A
29,913,110	29,913,111	Intron	SNV	Het	A	A/G	A/G	A/G	A/G	A/G	A/G

Table S8. Re-sequencing to verify CDS intra-CEH variants.

Forward Primer	Reverse Primer	Position	Variant Type	Effect	Gene	Exon	Ref	B46BM		B46ZS		B46CM	
								CG	PCR	CG	PCR	CG	PCR
CTTCAGGGCTAA GCTCAGGG	AGTTGCATGGAGC TGCTAGG	29634003	SNV	missense	MOG	3	G	G/G	G/G	C/C	C/C	G/G	G/G
CAGTTCCATCTTC CCCACCCAC	GTCTCTGGGACA AGGGACCTG	32190484	SNV	synonymous	NOTCH4	3	A	A/G	A/G	G/G	G/G	A/G	A/G

Forward Primer	Reverse Primer	Position	Variant Type	Effect	Gene	Exon	Ref	B58AL		B58SC		B58CM	
								CG	PCR	CG	PCR	CG	PCR
AGCAGTCACAAGT CACAGGG	CAGCCCATCGCA TGCTCAAT	29913037	SNV	missense	HLA-A	7	G	A/G	A/G	A/G	A/G	A/G	A/G

Table S9. Novel SNVs in the A33-B58-DR3 haplotype.

Position Start	Position End	Alternate Allele	Reference Allele	Gene
28,840,256	28,840,257	C	A	LOC401242 Downstream
29,035,463	29,035,464	G	A	LOC100129636 intron
29,035,477	29,035,478	T	C	LOC100129636 intron
29,734,245	29,734,246	T	G	IFITM4P Downstream
29,747,779	29,747,780	A	C	IFITM4P Downstream
29,765,233	29,765,234	C	A	LOC554223 3' UTR
29,779,058	29,779,059	G	A	HLA-G Downstream
29,779,067	29,779,068	G	A	HLA-G Downstream
29,783,925	29,783,926	C	T	HLA-H Upstream
29,783,930	29,783,931	T	C	HLA-H Upstream
29,783,931	29,783,932	A	C	HLA-H Upstream
29,784,433	29,784,434	C	T	HLA-H Upstream
29,859,618	29,859,619	A	C	HLA-H Downstream
29,859,620	29,859,621	C	T	HLA-H Downstream
29,859,927	29,859,928	G	A	HLA-H Downstream
29,859,928	29,859,929	T	C	HLA-H Downstream
29,867,025	29,867,026	T	A	HLA-H Downstream
29,867,026	29,867,027	G	C	HLA-H Downstream
29,869,426	29,869,427	G	A	HLA-H Downstream
29,869,429	29,869,430	A	C	HLA-H Downstream
29,869,434	29,869,435	C	T	HLA-H Downstream
29,872,187	29,872,188	G	A	HCG4B Upstream
29,872,188	29,872,189	A	G	HCG4B Upstream
29,872,841	29,872,842	A	T	HCG4B Upstream
29,872,843	29,872,844	A	T	HCG4B Upstream
29,890,605	29,890,606	A	C	HCG4B Upstream
29,903,515	29,903,516	G	C	HLA-A Upstream
29,904,860	29,904,861	T	C	HLA-A Upstream
29,906,004	29,906,005	A	C	HLA-A Upstream
29,906,078	29,906,079	C	A	HLA-A Upstream
29,906,079	29,906,080	T	C	HLA-A Upstream
29,906,654	29,906,655	T	C	HLA-A Upstream
29,906,674	29,906,675	C	T	HLA-A Upstream
29,909,682	29,909,683	C	T	HLA-A Upstream
29,909,685	29,909,686	T	C	HLA-A Upstream
29,909,688	29,909,689	A	C	HLA-A Upstream
29,909,834	29,909,835	G	A	HLA-A Upstream
29,913,273	29,913,274	C	T	HLA-A 3' UTR
29,913,837	29,913,838	C	G	HLA-A Downstream
29,913,944	29,913,945	G	A	HLA-A Downstream
29,914,625	29,914,626	C	T	HLA-A Downstream
29,914,943	29,914,944	C	T	HLA-A Downstream
29,914,944	29,914,945	T	G	HLA-A Downstream
29,915,123	29,915,124	A	C	HLA-A Downstream
29,915,147	29,915,148	T	C	HLA-A Downstream
29,915,148	29,915,149	G	A	HLA-A Downstream
29,915,150	29,915,151	G	T	HLA-A Downstream
29,915,966	29,915,967	T	C	HLA-A Downstream
29,926,156	29,926,157	A	G	HCG9 Upstream
29,944,177	29,944,178	A	C	HCG9 intron
29,944,307	29,944,308	G	T	HCG9 intron
29,944,308	29,944,309	G	C	HCG9 intron
29,944,324	29,944,325	A	T	HCG9 intron
29,944,326	29,944,327	G	A	HCG9 intron
29,944,327	29,944,328	C	G	HCG9 intron
29,955,808	29,955,809	C	T	HCG9 Downstream
29,956,118	29,956,119	G	A	HCG9 Downstream
29,956,126	29,956,127	C	G	HCG9 Downstream
29,956,134	29,956,135	C	A	HCG9 Downstream
29,956,137	29,956,138	C	T	HCG9 Downstream
29,956,138	29,956,139	A	G	HCG9 Downstream
29,956,484	29,956,485	C	T	HCG9 Downstream
29,956,486	29,956,487	G	T	HCG9 Downstream
29,960,539	29,960,540	C	T	HLA-J Upstream
29,960,552	29,960,553	A	T	HLA-J Upstream
29,960,564	29,960,565	T	C	HLA-J Upstream
29,967,872	29,967,873	T	C	HLA-J Upstream
29,968,000	29,968,001	A	C	HLA-J Upstream
29,968,002	29,968,003	C	G	HLA-J Upstream
29,968,011	29,968,012	G	A	HLA-J Upstream
29,968,015	29,968,016	C	G	HLA-J Upstream
30,467,075	30,467,076	A	G	HLA-E Downstream
30,467,077	30,467,078	C	G	HLA-E Downstream
30,467,086	30,467,087	G	A	HLA-E Downstream
31,348,653	31,348,654	G	A	HLA-E Downstream
31,348,655	31,348,656	T	G	HLA-E Downstream
31,363,403	31,363,404	T	A	MICA Upstream

Table S10. Novel SNVs in the A2-B46-DR9 haplotype.

Position Start	Position End	Alternate Allele	Reference Allele	Gene
29,632317	29,632,318	T	C	MOG intron
29,634,002	29,634,003	C	G	MOG exon 3
29,635,507	29,635,508	A	G	MOG intron
29,642,911	29,642,912	C	G	ZFP57 intron
29,645,725	29,645,726	T	C	ZFP57 Downstream
29,650,130	29,650,131	G	A	ZFP57 Downstream
29,654,569	29,654,570	A	T	ZFP57 Downstream
29,665,318	29,665,319	T	C	ZFP57 Downstream
29,665,360	29,665,361	G	A	ZFP57 Downstream
29,666,307	29,666,308	G	A	ZFP57 Downstream
29,667,478	29,667,479	T	C	ZFP57 Downstream
29,670,365	29,670,366	T	C	HLA-F Upstream
29,670,442	29,670,443	A	G	HLA-F Upstream
29,671,092	29,671,093	A	G	HLA-F Upstream
29,671,299	29,671,300	T	G	HLA-F Upstream
29,671,769	29,671,770	A	C	HLA-F Upstream
29,672,940	29,672,941	C	T	HLA-F Upstream
29,673,817	29,673,818	C	T	HLA-F Upstream
29,673,928	29,673,929	A	G	HLA-F Upstream
29,676,316	29,676,317	A	G	HLA-F Upstream
29,677,099	29,677,100	A	G	HLA-F Upstream
29,677,640	29,677,641	C	T	HLA-F Upstream
29,677,786	29,677,787	T	C	HLA-F Upstream
29,682,866	29,682,867	C	T	HLA-F Upstream
29,683,808	29,683,809	T	C	HLA-F Upstream

Position Start	Position End	Alternate Allele	Reference Allele	Gene
29,717,380	29,717381	G	C	HLA-F-AS1 Downstream
29,770,566	29,770,567	T	C	LOC554223 Downstream
29,855,724	29,855,725	T	C	HLA-H intron
29,855,725	29,855,726	C	T	HLA-H intron
29,914,696	29,914,697	A	C	HLA-A Downstream
29,914,943	29,914,944	C	T	HLA-A Downstream
29,914,944	29,914,945	T	G	HLA-A Downstream
29,914,968	29,914,969	A	C	HLA-A Downstream
29,915,112	29,915,113	T	A	HLA-A Downstream
29,915,118	29,915,119	G	A	HLA-A Downstream
29,923,565	29,923,566	C	A	HLA-A Downstream
30,100,792	30,100,793	T	C	TRIM40 Upstream
30,570,769	30,570,770	T	C	PPP1R10 intron
30,768,863	30,768,864	C	T	IER3 Downstream
30,789,947	30,789,948	T	C	DDR1 Upstream
30,981,052	30,981,053	G	A	MUC22 intron
30,981,055	30,981,056	A	G	MUC22 intron
30,981,057	30,981,058	T	C	MUC22 intron
30,981,064	30,981,065	G	A	MUC22 intron
31,197,816	31,197,817	C	T	HLA-C Upstream
31,197,818	31,197,819	T	C	HLA-C Upstream
31,197,820	31,197,821	C	T	HLA-C Upstream
31,496,777	31,496,778	G	C	MCCD1 5' UTR
31,850,914	31,850,915	A	G	EHMT2 intron
32,050,846	32,050,847	T	C	TNXB intron

Table S11. Regions of low and high variation between the sequenced MHC haplotypes.

Regions with variations below genome average				
Start	End	Length (kb)	Mean variant /5kb	Genes
28350000	28415000	65	0.61	ZSCAN12, ZSCAN23
28430000	28495000	65	1.96	GPX6
28560000	28670000	110	0.78	Downstream SCAND3, Upstream LOC401242
29545000	29630000	85	1.47	SNORD32B, OR2H2, GABBR1
30545000	30680000	135	1.49	PPP1R10, MRPS18B, ATAT1, C6orf136, DHX16, PPP1R18, NRM
31690000	31805000	115	1.66	DDAH2, CLIC1, MSH5, SAPCD1, VWA7, VARS, LSM2, HSPA1L, HSPA1A, HSPA1B
31845000	31895000	50	1.90	EHMT2, C2, ZBTB12
31905000	31965000	60	1.39	C2, CFB, NELFE, SKIV2L, DOM3Z, STK19, C4A, C4B
31980000	32160000	180	1.84	CYP21A2, TNXB, ATF6B, FKBPL, PRRT1, LOC100507547, PPT2, EGFL8, AGPAT1, RNF5, AGER,PBX2
32855000	32895000	40	1.35	LOC100294145

Regions with extreme variation				
Start	End	Length (kb)	Mean variant /5kb	Genes
29665000	29715000	50	16.16	HLA-F
29730000	29805000	75	26.54	HCG4, LOC554223, HLA-G
29820000	29975000	155	36.93	HLA-H, HCG4B, HLA-A, HCG9
31005000	31105000	100	19.59	HCG22, C6orf15, PSORS1C1, CDSN
31155000	31360000	205	45.62	HCG27, HLA-C, HLA-B
31375000	31415000	40	22.38	MICA
32190000	32220000	30	18.97	Downstream NOTCH4
32665000	32775000	110	37.40	HLA-DQA2, HLA-DQB2

Table S12. Prime pairs to amplify genomic fragment carrying specific alleles of each intra-CEH variant s for cloning into expression vector.

rsID	Forward Primer	Reverse Primer
rs3117318	CTTTTTGTGTTTTTAGTAGAG**(G/A)**TGGGGATTCACCTTGTTGGTC	TCGAGACCAACAAGGTGAATCCCCA**(C/T)**CTCTACTAAAAACACAAAAAGAGCT
rs3129073	CTATGTCATCCACAAAGTTCT**(G/A**)TCACCCTATTCCATAGCATAC	TCGAGTATGCTATGGAATAGGGTGA**(C/T)**AGAACTTTGTGGATGACATAGAGCT
rs3095271	CGAGTAGCTGGGATTACAGGC**(A/G)**CACGCCACCATGCCCAGCTAC	TCGAGTAGCTGGGCATGGTGGCGTG**(T/C)**GCCTGTAATCCCAGCTACTCGAGCT
rs29228	CTACGCCCATCTCGGACAAAA**(A/G)**CAGAAATGTATGTCTCAGTTC	TCGAGAACTGAGACATACATTTCTG**(T/C)**TTTTGTCCGAGATGGGCGTAGAGCT
rs3095294	CGGCCCAGCGCCGTGGCTCAC**(A/G)**CCTGTAATCCCAGCACTGTGC	TCGAGCACAGTGCTGGGATTACAGG**(T/C)**GTGAGCCACGGCGCTGGGCCGAGCT
rs3129069	CAGATCACAAGGTCAGGAGAT**(T/C)**GAGACCATCCTGGCTAAAACC	TCGAGGTTTTAGCCAGGATGGTCTC**(A/G)**ATCTCCTGACCTTGTGATCTGAGCT
rs3130251	CGCGGTGGCTCACACCTATAA**(C/T)**CCCAAAACTTTGGGAGGCCGC	TCGAGCGGCCTCCCAAAGTTTTGGG**(G/A)**TTATAGGTGTGAGCCACCGCGAGCT
rs3095290	CCCCAGCACTTTGGGAGGCC**(A/G)**AGGCCAGCGGATCACCTGAGGC	TCGAGCCTCAGGTGATCCGCTGGCCT**(T/C)**GGCCTCCCAAAGTGCTGGGGAGCT
rs374317	CAGAAAGGAAAGGAGGCCGGA**(T/C)**GCGGTGGCTCACGCCTGTAAC	TCGAGTTACAGGCGTGAGCCACCGC**(A/G)**TCCGGCCTCCTTTCCTTTCTGAGCT
rs375248	CGTGAGCCAAGATCGCGCCAC**(T/C)**GCACTCCAGCCTGGGTGACAC	TCGAGTGTCACCCAGGCTGGAGTGC**(A/G)**GTGGCGCGATCTTGGCTCACGAGCT
rs3094579	CGGGTGACAGAGCGAGACTCC**(A/G)**TCTCAAAAAAAAAAAAAAGAC	TCGAGTCTTTTTTTTTTTTTTGAGA**(T/C)**GGAGTCTCGCTCTGTCACCCGAGCT
rs375984	CTGAAGGATGCCAGGATGGTG**(T/C)**GGGAGATGGAGAAAGGTCTTC	TCGAGAAGACCTTTCTCCATCTCCC**(A/G)**CACCATCCTGGCATCCTTCAGAGCT
rs376319	CAGTCCAGGACCTGGACCCCA**(T/C**)CTCTCTCTAGCTTAGTCTCCC	TCGAGGGAGACTAAGCTAGAGAGAG**(A/G)**TGGGGTCCAGGTCCTGGACTGAGCT
rs2535238	CTCCCTCAAAGCTGGGCCAC**(A/C)**GAGTTCAGGGCCCTGGTCACCC	TCGAGGGTGACCAGGGCCCTGAACTC**(T/G)**GTGGCCCAGCTTTGAGGGAGAGCT
rs2747421	CGCCTGGGCTAGGTGTCTGCC**(C/G)**TCTGTTCTACCCTGCTTCTAC	TCGAGTAGAAGCAGGGTAGAACAGA**(G/C)**GGCAGACACCTAGCCCAGGCGAGCT
rs3129063	CTCAATACATGTAGTAGTTCC**(A/C)**CTTTATCATGGTTTTGCTTTC	TCGAGAAAGCAAAACCATGATAAAG**(T/G)**GGAACTACTACATGTATTGAGAGCT
rs387642	CCTTTATCATGGTTTTGCTTT**(T/C)**CAATGCTTCAGTTACCCATGC	TCGAGCATGGGTAACTGAAGCATTG**(A/G)**AAAGCAAAACCATGATAAAGGAGCT
rs387640	CGCAGATCATCTGAGGTCAGG**(G/A)**GTTCGAGATCAGCCTGGTCAC	TCGAGTGACCAGGCTGATCTCGAAC**(C/T)**CCTGACCTCAGATGATCTGCGAGCT
rs3129062	CGTTGCCATGAGCCAAGACTG**(T/C)**GCCACTGCACTCCAGCCTGGC	TCGAGCCAGGCTGGAGTGCAGTGGC**(A/G)**CAGTCTTGGCTCATGGCAACGAGCT
rs448489	CTTGCACTCCAGCCAGGGAAA**(C/T)**GAGAGTGAAACTCCGTCTCAC	TCGAGTGAGACGGAGTTTCACTCTC**(G/A)**TTTCCCTGGCTGGAGTGCAAGAGCT
rs2747427	CACCCAAAACCCTTTATCCAC**(G/A)**GCAATTCTGAGAATGATGAGC	TCGAGCTCATCATTCTCAGAATTGC**(C/T)**GTGGATAAAGGGTTTTGGGTGAGCT
rs2747428	CACCCTTTATCCACAGCAATT**(G/C)**TGAGAATGATGAGAATCCCCC	TCGAGGGGGATTCTCATCATTCTCA**(C/G)**AATTGCTGTGGATAAAGGGTGAGCT
rs417764	CACAGCAATTCTGAGAATGAT**(A/G)**AGAATCCCCCTCACCCCTCAC	TCGAGTGAGGGGTGAGGGGGATTCT**(T/C)**ATCATTCTCAGAATTGCTGTGAGCT
rs3129058	CTTAGTGGGATTCACCCTTGT**(T/C)**GTCACCAACCCTGCTACTCCC	TCGAGGGAGTAGCAGGGTTGGTGAC**(A/G)**ACAAGGGTGAATCCCACTAAGAGCT
rs2747429	CGGGCGCTCCTGGCCCTGTCC**(C/T)**CCGCGCTTAGTTTGTCATTGC	TCGAGCAATGACAAACTAAGCGCGG**(G/A)**GGACAGGGCCAGGAGCGCCCGAGCT
rs416568	CCGGAGATCGCGCCATTGCAC**(A/T)**CCAGCCAGGGAAATGAGAGTC	TCGAGACTCTCATTTCCCTGGCTGG**(T/A)**GTGCAATGGCGCGATCTCCGGAGCT
rs365052	CCCGCGCTTAGTTTGTCATTG**(C/G)**GCGCCCAGATCCGGAACCCCC	TCGAGGGGGTTCCGGATCTGGGCGC**(G/C)**CAATGACAAACTAAGCGCGGGAGCT
rs2747431	CTACCTTCCCCGATGCCCATC**(T/C)**GCCCAGAATCTTCCTTCTGGC	TCGAGCCAGAAGGAAGATTCTGGGC**(A/G)**GATGGGCATCGGGGAAGGTAGAGCT
rs3117295	CTTCCTTTCTATGCCCCTTTA**(C/T)**CTCTAATGTCCCTATTCTGCC	TCGAGGCAGAATAGGGACATTAGAG**(G/A)**TAAAGGGGCATAGAAAGGAAGAGCT
rs7738158	CCCCGTCTCACTCTCCCAAGT**(A/G)**GCTGGAATCACTGGCGCATGC	TCGAGCATGCGCCAGTGATTCCAGC**(T/C)**ACTTGGGAGAGTGAGACGGGGAGCT
rs3129056	CCACTGTTTTGGCCTGGAGGT**(G/A)**TCTCTTCCCTAAAGCCAAAAC	TCGAGTTTTGGCTTTAGGGAAGAGA**(C/T)**ACCTCCAGGCCAAAACAGTGGAGCT
rs3129048	CGCAGACGGGGCTGAACCTGA**(A/G)**GTCAACCACGCTGAGACGGAC	TCGAGTCCGTCTCAGCGTGGTTGAC**(T/C)**TCAGGTTCAGCCCCGTCTGCGAGCT
rs3129047	CTGAGGTCAACCACGCTGAGA**(T/C)**GGAGGTTCCTCCTGAGCACCC	TCGAGGGTGCTCAGGAGGAACCTCC**(A/G)**TCTCAGCGTGGTTGACCTCAGAGCT
rs1610588	CGCCCAGAAACCCATGAATAG**(T/C)**GTCCTTGGGCTGACCTATGCC	TCGAGGCATAGGTCAGCCCAAGGAC**(A/G)**CTATTCATGGGTTTCTGGGCGAGCT
rs1610589	CACCTGCTCATCTGCATGGCC**(A/G)**TGTGCCTGACATGGTCAGAAC	TCGAGTTCTGACCATGTCAGGCACA**(T/C)**GGCCATGCAGATGAGCAGGTGAGCT

Table S13. RCCX modular structure in 13 reference human cell lines.

Cell line	RCCX Structure	C4A	C4B	C4L	C4S	TNXA
COX	Monomodular	0	2	0	2	0
QBL		2	0	2	0	0
MOU		0	2	2	0	0
PGF	Bimodular	2	2	4	0	2
SSTO		2	2	4	0	2
DBB		2	1	2	2	2
WT51		4	0	4	0	2
MADURA		2	2	0	4	2
CB6B	Quadrimodular	4	4	6	2	6
WT8	Heterozygous	1	2	3	0	1
DAUDI		2	1	2	1	1
MANIKA		2	3	2	3	3
HOM		3	2	4	1	3
